# Cholinergic modulation of neural networks supports sequential and complementary roles for NREM and REM states in memory consolidation

**DOI:** 10.1371/journal.pcbi.1013097

**Published:** 2025-06-17

**Authors:** Michael Satchell, Edith Butel-Fry, Zahraa Noureddine, Alexis Simmons, Nicolette Ognjanovski, Sara J. Aton, Michal R. Zochowski

**Affiliations:** 1 Department of Physics, University of Michigan, Ann Arbor, Michigan, United States of America; 2 Department of Physics, Baylor University, Waco, Texas, United States of America; 3 Department of Molecular, Cellular and Developmental Biology, University of Michigan, Ann Arbor, Michigan, United States of America; 4 Biophysics Program, University of Michigan, Ann Arbor, Michigan, United States of America; University College London, UNITED KINGDOM OF GREAT BRITAIN AND NORTHERN IRELAND

## Abstract

Across vertebrate species, sleep consists of repeating cycles of NREM followed by REM. However, their respective functions, and their stereotypic cycling pattern are not well understood. Using a simplified biophysical network model, we investigate the potential role of cholinergic modulation, acting via the muscarinic receptors, on network dynamics and memory consolidation. We show that low and high cholinergic levels associated with NREM and REM sleep, respectively, may play critical, sequential roles in memory consolidation. The network dynamics that facilitate these roles arise through alteration of neural excitability and changes to network-wide excitatory/inhibitory balance. At low acetylcholine (ACh) levels, reduced activation of inhibitory neurons leads to network-wide disinhibition and bursts of synchronized activity led by engram neurons, driving recruitment of additional excitatory neurons into the engram. In contrast, at high ACh levels, increased network inhibition suppresses firing in all but the most strongly recruited excitatory neurons, pruning the expanded engram population. Together, these results provide a testable hypothesis regarding the role of sleep state-specific cholinergic modulation in the process of memory consolidation.

## Author summary

Across animal species, sleep stereotypically is composed of non-REM (non-rapid eye movement, NREM) and REM (rapid eye movement) phases, with NREM preceding REM. It remains unclear what differential functions those two phases play in sleep-dependent memory consolidation – a process leading to better recollection of recently experienced events. Using a simplified computational model combined with analysis of *in vivo* recordings from mouse hippocampus, we show that changes in acetylcholine levels during NREM and REM sleep may drive different roles in memory consolidation. Specifically, we show that low levels of acetylcholine during NREM disinhibit activity of otherwise quiescent subpopulations of excitatory neurons permitting the expansion of memory traces. In contrast, high levels of acetylcholine during REM create a more competitive environment for neuronal activity leading to inhibition of less driven cells and preventing an overlap in memory representations when more than one memory trace is involved. We further demonstrate advantages of the physiological structure of sleep in aiding this process.

## Introduction

In vertebrate species, sequential cycling from wake to non-rapid eye movement (NREM) sleep to REM sleep is a universal pattern [1]. The evolutionary origins of the physiologically distinct NREM and REM states are a matter of speculation [2]. However, available data suggest that both are essential for brain functions, including long-term memory consolidation and learning-associated synaptic plasticity [2–5]. The two states have dramatically different features – including characteristic neuronal firing patterns and rate changes, network oscillations, and neuromodulation [2–4,6–11] – suggesting that NREM and REM likely have distinct effects on brain circuits in relation to memory storage. Recent studies have identified molecular [12,13] and electrophysiological [14–24] changes in neural circuits during post-learning NREM and REM sleep. There are number of neurochemical changes differentiating sleep stages [25]. One of the most prominent physiological variables distinguishing NREM and REM sleep is the brain-wide level of acetylcholine (ACh), dropping to extremely low concentrations in NREM and rising to especially high concentrations in REM [25,26]. Cholinergic projections are found throughout the forebrain, midbrain and brainstem [27]. ACh regulates neural excitability and is essential for brain processes ranging from sleep-wake regulation to sensory detection [27–29].

In the hippocampus, cholinergic inputs originating from the medial septum (MS) [30,31] provide direct input to both principal neurons and interneurons [32]. The septo-hippocampal projection is a critical regulator of hippocampal function, regulating theta oscillations vital in memory acquisition and storage [33]. ACh acts through nicotinic and muscarinic receptors [34,35] with fast and slow neuromodulatory effects, respectively. There are five subtypes of muscarinic receptors, M1 through M5 [36]. The M1 receptor is the most abundant muscarinic ACh receptor in the brain and is especially prominent in the neocortex and hippocampus [37,38]. M1 receptors regulate the excitability of hippocampal neurons [39,40] via coupling to slow, non-inactivating potassium channels. The current through these channels, termed “M-current”, is hyperpolarizing and is blocked by ACh binding to the M1 receptor. Increasing ACh concentration reduces M-current and increases neuronal excitability, while low levels of ACh leave this hyperpolarizing channel open [39]. In addition to its effects on the M-current, ACh also causes a reduction in the afterhyperpolarization (AHP) calcium-dependent potassium current, leading to a reduction in spike frequency accommodation [41,42]. This increase in cellular excitability due to ACh is similar to the effect of ACh on excitability through the M-current, so incorporation of the AHP current into our model would not fundamentally alter the population dynamics or balance of inhibition that are crucial to our results. Thus, we chose to focus our model on the role ACh plays in modulating the M-current. Changing from low to high ACh concentration shifts neurons from Type 2 to Type 1 excitability states. This shift dramatically changes their firing frequency responses to input current (i.e., their F-I curve or gain function) [43]. The F-I curve of Type 1 neurons is continuous with a steep slope, while the Type 2 F-I curve is discontinuous at the transition from quiescence to spiking with a more gradual slope [43]. Another difference between Type 1 and Type 2 dynamics is a qualitative distinction in spike timing as a response to weak excitatory input – the phase response curve (PRC) of a neuron [44]. A Type 1 PRC is uniformly positive, meaning that an excitatory input always advances the timing of the next action potential. Type 2 dynamics are characterized by a biphasic PRC, with spike timing delays induced when input arrives shortly after a previous action potential, and spike timing advances only induced at longer intervals [43,44]. The biphasic nature of neurons with Type 2 PRCs (under low ACh, high M-current conditions) facilitates spike synchrony between neurons interacting within a network [43,45]. Thus, ACh modulation through M1 receptors can dramatically change neural activity at the individual neuron and network level, as observed experimentally [46].

At the network level, ACh may also alter the excitatory-inhibitory balance in brain networks [47,48]. In the hippocampus, ACh has the ability to selectively activate somatostatin-expressing (SST+) interneurons [48–53], which can affect the encoding, storage, and recall of memories. Inhibitory gating of activity in hippocampal dentate gyrus (DG) by SST+ interneurons in the hours following learning constrains memory consolidation [13]. Chemogenetic suppression of medial septal ACh projections to hippocampus after single-trial fear conditioning improves sleep-dependent fear memory consolidation and increases DG granule cell activity. Conversely, chemogenetic activation of ACh inputs suppresses DG granule cell activity and impairs memory consolidation [13]. These results are consistent with prior research indicating that suppression of cholinergic signaling after learning benefits memory consolidation [54,55]. The ACh M1 receptor is specifically implicated in memory consolidation [56,57].

Together, these findings suggest that the NREM-associated decrease in ACh transmission is an essential feature for sleep-dependent memory consolidation. However, there are accumulating data suggesting that REM – a high-ACh brain state - also plays an important role in consolidation of hippocampus-dependent memories [58–62]. It remains unclear what roles NREM and REM play in the storage of information in neural networks. The influence of ACh on neural dynamics may be key to understanding the roles that NREM and REM sleep play in memory storage.

Here, using *in silico* models, we investigate how ACh-regulated mechanisms alter memory engrams in a neural network, in the context of sleep-dependent memory consolidation. We simulate changes in the concentration of ACh through the specific cholinergic effects on the M-current as a proxy to model the effects of the states of NREM and REM on a neural circuit. We show that with reduced ACh (referred to as the ACh^–^ state) during NREM, disinhibition and an increased ability of principal neurons to fire in synchrony recruits new neurons into recently formed engrams. In contrast, high ACh (referred to as the ACh^+^ state) during REM leads to increased activation of inhibitory interneurons, driving network dynamics that promote competitive and selective pruning of the engram neuron population [63]. We further show that the ACh^+^ state is particularly important when multiple memory engrams must be consolidated simultaneously, as it reduces overlap between engram representations. Repeated iterations of the ACh^–^ →ACh^+^ cycle (such as occurs across a night of sleep) results in the optimal expansion and segregation of engrams in the network. Reversal of the ACh^–^ →ACh^+^ (NREM→REM) sequence by instead first entering the ACh^+^ (REM) state fails to recruit new neurons into the engrams, preventing engram expansion [64]. Together, these data suggest that ACh-regulated network dynamics during NREM and REM play distinct and complementary roles in memory consolidation, with NREM→REM sequential state ordering being an essential feature.

## Results

### Distinct network activity patterns are induced by ACh^–^ and ACh^+^ states

The hippocampal principal neuron population is heterogeneous with respect to firing rate [65]. In the context of *de novo* memory formation, a subset of memory-encoding “engram” neurons within the hippocampal network become more active, maintain a higher level of activity over time [66], and increase the strength of functional connections to one another [67]. To model effects of NREM and REM sleep on recently encoded hippocampal neurons, we constructed a reduced neural network composed of two excitatory cell populations: 1) a highly active memory encoding population representative of a single memory (“engram backbone”, EB population, 40 neurons) and 2) sparsely firing neurons with plastic connections from the EB cells (SF population, 80 neurons). These excitatory neurons form a recurrent network with ~10%, primarily random connectivity (see Methods), reminiscent of the hippocampal region CA3 [68]. We found that similar results were obtained when this reduced model was scaled up to 540 (total) neurons (S1 Fig). To model changes to the network initial memory engram formation, EB neurons’ connections to other EB neurons were selectively strengthened [64,69]. With the exception of synaptic connections between EB cells (which are assumed to have strengthened maximally during engram formation), all other excitatory synapses are subject to spike-timing dependent synaptic plasticity (STDP). Interneurons within the network inhibited both EB and SF neurons. To mimic connectivity within forebrain structures, this interneuron population was numerically smaller (20 neurons), but with a high connection density to the entire network (50%). Consistent with available data [66,70–73], these interneurons differentially targeted EB and SF populations, providing a higher level of inhibition to SF neurons via both fast (GABA_A_-mediated) and slow (GABA_B_ - mediated) receptor kinetics (see Methods). While this led to initial differences in firing rate between EB and SF neurons, our results are not dependent upon this aspect of network connectivity, as similar network activity was achieved with equal inhibition to the two populations, when intrinsic current modulated their excitability instead – see supplemental data S2 Fig). To model effects of state-specific neuromodulation, all three populations were simultaneously influenced by changing ACh levels, modeled as a change in the slow, hyperpolarizing M-current conductance (see Methods) [74]. The low ACh neuromodulation during NREM sleep was modeled by high M-current conductance ($g_{Ks}=1.5\frac{mS}{cm^{2}}$, ACh^–^), while higher, REM-associated ACh release was modeled by low M-current conductance ($g_{Ks}=0\frac{mS}{cm^{2}}$, ACh^+^).

**Fig 1:**
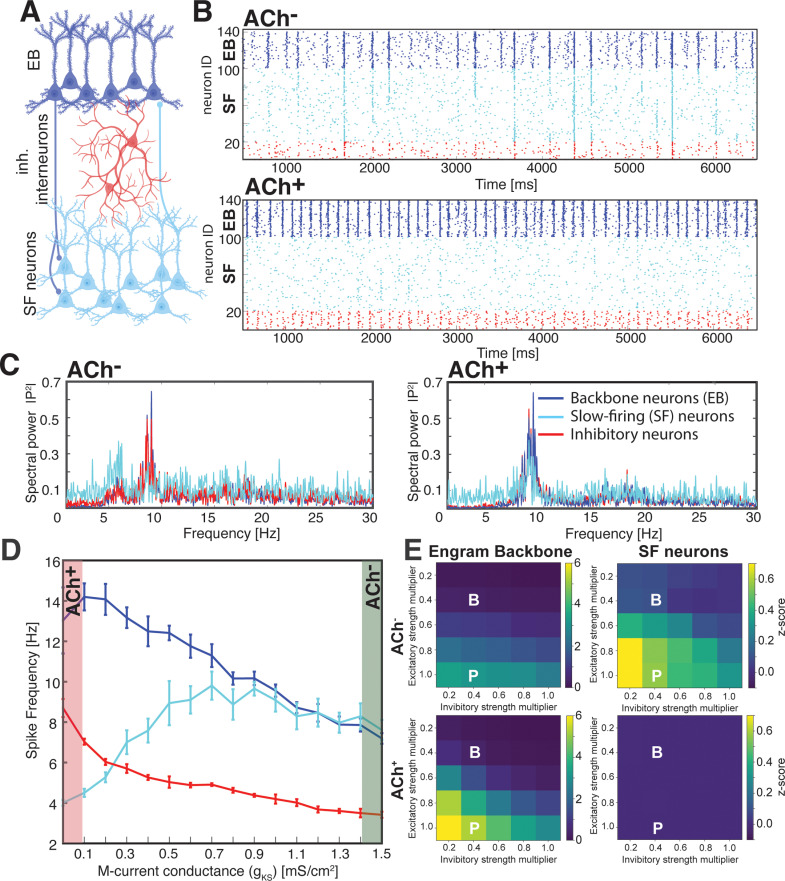
ACh-regulated current modulates activation of neuron populations during ACh^–^ and ACh ^+^ network states. *A)* Schematic of the model network, which consists of an excitatory, memory-encoding engram “backbone” (EB) neuron population (***dark blue***), inhibitory interneurons (***red***), and an excitatory population that is less excitable (SF) and non-memory-encoding (***teal***). ***B)*** Representative raster plot of firing patterns for the three populations during a NREM-like ACh^–^ state (high g_Ks_; **top**) and a REM-like ACh^+^ state (high ACh, low g_Ks_; **bottom**). ***C)*** Spectral analysis of firing patterns during ACh^–^ (**top**) and ACh^+^ (**bottom**) states. Data are shown for firing of the entire network (**left, *black line***) and separately for each neuron population (***colors denoted as above***). ***D)*** Mean firing frequency for the three network populations as a function of maximal g_Ks_ conductance. High g_Ks_ conductance during ACh^–^ (highlighted in ***green***) causes disinhibition of the SF population, and permits their participation in bursts driven by EB neurons. Low g_Ks_ conductance during ACh^+^ (highlighted in ***red***) increases activity in backbone and inhibitory neurons, while SF neuron activity is suppressed. Values indicate mean ± SEM, for 4 simulation runs. **E)** Mean functional connectivity between pairs of EB neurons (**left**) and between pairs of SF neurons (**right**), as a function of strength of EB neurons’ connections to both other SF neurons and inhibitory interneurons. The default connection strength (see **Methods**) was multiplied by a factor as indicated on the Y and X axis, respectively. Mean functional connectivity varies more with variation in synaptic strength in the ACh^+^ vs. ACh^–^ state.

We first investigated population-level activity dynamics as a function of M-current conductance (Fig 1) for a network with a single encoded memory (single EB population; Fig 1A). Mean spike frequency for neurons within each population (EB neurons, SF neurons and inhibitory interneurons) was measured for $g_{Ks}$ values between 0 and 1.5$\frac{mS}{cm^{2}}$. At low ACh levels ($g_{Ks}=1.5\frac{mS}{cm^{2}}$) mimicking NREM conditions (ACh^–^), the I-F curve of individual neurons was flattened due to reduced excitability, lowering their firing frequency in response to input current [43,75]. We also observed heterogeneous responses among cell populations in the network, with the firing frequency of both inhibitory interneurons and EB neurons (Fig 1D, dark blue and red lines) decreasing. Lower firing rates among interneurons resulted in disinhibition of SF neurons, increasing their firing rate (Fig 1D, teal line). As a result, during the ACh^–^ state SF neurons are able to be recruited into spontaneous bursts of network activity. These bursts emerge as a result of low network inhibition, Type 2 neural excitability, and biphasic PRCs among neurons in the network [76,77] (Fig 1B, top; 1C, left). Rhythmic firing in the network in this low-ACh state is strongly influenced by the membrane resonance properties associated with Type 2 excitability, and less so by the time constants of inhibitory signaling. At high ACh levels ($g_{Ks}=0\frac{mS}{cm^{2}}$) mimicking REM conditions (ACh^+^), both EB neurons and interneurons exhibited periodic bursts of activity, but at a higher frequency (Fig 1B bottom, 1C right panel). This periodic bursting resulted from slow-decaying inhibitory currents (GABA_B_) in EB neurons (reminiscent of a slow version of the mechanism underlying gamma activity [76,77]). In an oscillatory mechanism distinct from the dynamics of the ACh^–^ state, strong activation of EB neurons in the ACh^+^ state recruits a heavy burst of firing among interneurons, which in turn synchronously inhibits all excitatory neurons until the slow inhibitory postsynaptic current decays, allowing excitatory neurons to spike again. Because interneurons are more active in the ACh^+^ condition and SF neurons are less able to self-excite than the strongly interconnected EB cell group, SF neurons remain relatively silent during this period of high inhibition (Fig 1D), with their firing driven solely by a random noise injected current (see Methods). These waves of excitation and inhibition manifest as network oscillations near theta frequency (Fig 1C, right), reminiscent of the theta rhythm seen in the hippocampus during REM sleep in rodents (and to a somewhat lesser extent in humans [78]). However, we note that while there is evidence that SST+ interneurons promote intrinsic theta-band oscillations in the hippocampus [79–82], the theta rhythm in the hippocampus is primarily understood to be driven by input from the medial septum, which we do not include in our model [83,84]. However, we believe that the source of the theta rhythm, as long as it is accompanied by an increase in inhibitory activity and ACh concentration, does not qualitatively alter our results. An additional observation from the model is a wider range of firing rates in the ACh^+^ state (Fig 1D), consistent with recent data showing that firing rates are more divergent among hippocampal neurons in REM sleep [85].

We next used a previously developed functional connectivity algorithm [86] to measure how the dynamics of firing in the ACh^–^ state and the ACh^+^ state affect network-wide functional connectivity. We first quantified changes to the mean functional connectivity between pairs of neurons by comparing pairwise spike timing relationships, either within the EB population or within the SF population (see Methods). We then systematically varied the strength of excitatory synapses from the EB population to both EB and SF neurons, and separately, EB excitatory synapses targeting inhibitory interneurons (Fig 1E). Strengthening synaptic connections from EB neurons to SF neurons (moving down the y-axis; Fig 1E) increased mean functional connectivity in the network, while strengthening backbone neurons’ synapses on inhibitory interneurons (moving along the x-axis from left to right; Fig 1E) decreased mean functional connectivity. However, these results differed between ACh^–^ and ACh^+^ states. We observed that within the EB population itself, functional connectivity was generally weaker during the NREM-like ACh^–^ state than in the REM-like ACh^+^ state, and did not vary as dramatically with incremental changes to synaptic strength. Conversely, mean functional connectivity between pairs of SF neurons was significantly higher during the ACh^–^ state than during the ACh^+^ state. Taken together, these findings suggest that both the strength of excitatory input from engram neurons and ACh-regulated network dynamics influence functional connectivity throughout the network.

### Modeled effects of learning and sleep states on functional connectivity are recapitulated in the mouse hippocampus following learning

**Fig 2:**
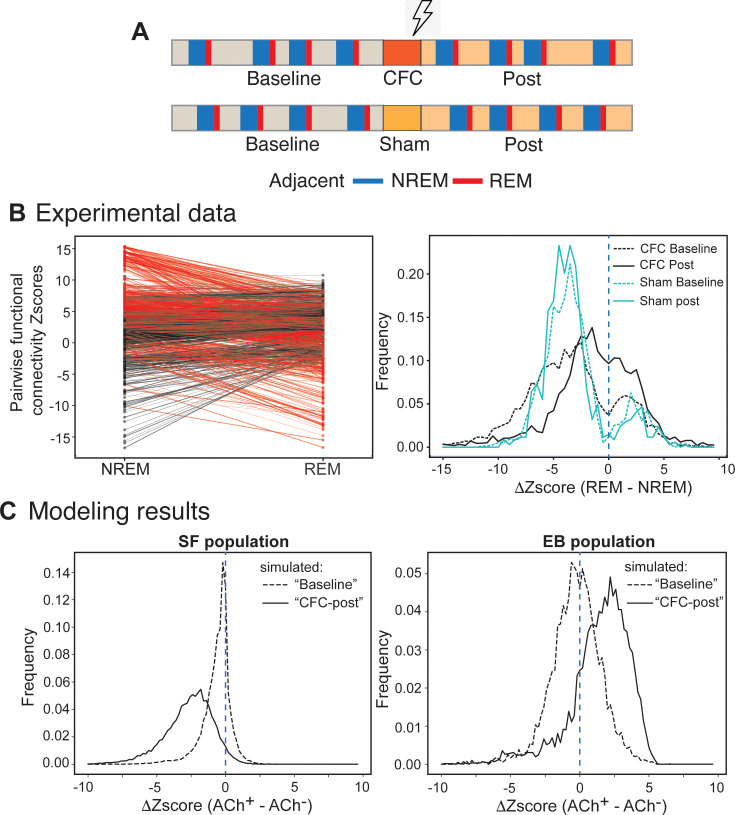
Changes in functional connectivity between NREM and REM – comparison of in vivo experimental and in silico modeling data. A) Schematic of analyzed experiments. (TOP, CFC) We analyzed 6h of recorded baseline when mouse is placed in home cage. Mouse is then placed in a new cage which it forages and at the end which the electric foot shock is applied (CFC). Mouse is then placed back in home cage - we analyze sleep bouts during 6h of post CFC recordings. (BOTTOM, SHAM) same as top, with the difference that electric shock is not applied during exposure to novel cage). Please refer to [14,16] for detailed description of experiments. **B) (Experiment)** Left panel - Change in functional connectivity (reported as change in z-scores, with |z| > 2) between adjacent NREM and REM sleep bouts (representative example of one animal). Red indicates pairs with decreasing functional connectivity from NREM to REM, black indicates pairs with increasing functional connectivity from NREM to REM. Right panel - baseline (dashed black line) and post-CFC (solid black line) changes for mice consolidating contextual fear memory, and baseline (dashed blue line) and post-sham (solid blue line) changes for control mice undergoing sham training. REM sleep generally weakens functional connectivity between principal neurons, relative to the preceding period of NREM. However, animals that underwent CFC exhibited a significant increase in the number of cell pairs that strengthen functional connectivity (during REM), as compared to baseline (K-S test comparing baseline and CFC *p* = 6.0102e-126). Such a change is absent for sham animals, as they exhibit a more modest change in the opposite direction (K-S test comparing baseline and sham *p* = 0.0014). These results indicate that during exposure to CFC, there is rapid network reorganization leading to more heterogenous interactions between populations of putative excitatory neurons, leading to selective strengthening of pairwise functional connectivity (i.e., recruitment) during REM – an effect observed to a much smaller extent during baseline and SHAM. The data shown in right panel is an average of experiments done on 5 animals undergoing CFC training and 5 Sham animals. **C) (Modeling Results)** Change in pairwise functional connectivity (reported as change in z-scores) between simulated ACh^–^ and ACh+ states, under conditions of either reduced (marked “B” on **Fig 1E** and referred to as baseline) or full strength of excitatory coupling (marked “P” on Fig 1E and referred to as CFC-post). Right panel - change in pairwise functional connectivity for baseline (dashed black line) and CFC-post (solid black line) for the SF population. Left panel – change in pairwise functional connectivity for the EB population. ACh^+^ dynamics causes broad pruning of weaker connections with the SF population, shifting the distribution towards lower functional connectivity values (lower z-scores) as compared to ACh^–^ state (center). Strong connections associated with the EB population are further strengthened in the ACh+ state.

To compare these *in silico* results with *in vivo* neural data, we measured pairwise functional connectivity among individual neurons recorded in mouse hippocampal region CA1 during sleep in the hours following single-trial contextual fear conditioning (CFC) [14,16] (Fig 2A top). Functional connectivity was compared between 6 h of baseline recording prior to CFC and the first 6 h post-CFC (a time window critical for sleep-dependent memory consolidation [14,16]) for adjacent bouts of naturally-occurring NREM and REM sleep (i.e., for consecutive bouts of NREM and REM sleep that were separated by less than 10s). An example of such a comparison is shown for a representative mouse in the left panel of Fig 2B. As a negative control, we also compared pairwise functional connectivity for sham-conditioned mice, in which no association between the context and a foot shock was made (“sham”; Fig 2A (bottom). For sham-conditioned mice, the underlying assumption is that in the absence of associative memory, no substantial engram neuron population with increased activity is present in the hippocampus. Consistent with our modeling results, we observed that average functional connectivity between pairs of putative principal (i.e., non-fast spiking) neurons was lower in REM as compared to NREM sleep across all recording conditions. However, following CFC, the REM-associated drop in functional connectivity was less pronounced than during the corresponding baseline period. Indeed, following CFC, many neuronal pairs showed stronger functional connectivity during REM than NREM – something rarely seen during baseline sleep (Fig 2B, solid and dashed black lines; KS test comparison of baseline and post-CFC sleep *p* < 0.0001). This learning-associated change in state-dependent functional connectivity was not observed in sham conditioned mice (Fig 2B, solid and dashed blue lines), which actually showed a slightly *greater* decline in functional connectivity across NREM→REM transitions after sham procedures (KS test comparison of baseline and post-sham *p* < 0.0014).

To better understand the mechanisms that could underlie these changes, we ran a similar analysis on simulated data using the network from Fig 1, comparing Z-scored pairwise functional connectivity changes between sequential ACh^–^ and ACh^+^ states. We made this measurement using two different excitatory connectivity strength values corresponding to connections originating from the EB population (as marked on Fig 1E). To simulate the “baseline” period before memory encoding, the EB neurons do not yet have their reciprocal connections (with weak EB synaptic outputs corresponding to the lack of a new memory trace; dashed lines on Fig 2C). For the “post-learning” simulations, EB neurons have their synapses set to a strengthened value (to represent the new memory trace present; solid lines on Fig 2C). Changes in functional connectivity across ACh^–^ and ACh^+^ were then measured separately for the EB and SF neuron populations. Both populations exhibited a modest drop in pairwise functional connectivity in the baseline condition from the ACh^–^ to the ACh^+^ state (Fig 2C) – similar to changes observed in mice between baseline NREM and REM states. In contrast, for the post-learning condition, the EB and SF groups exhibit dramatically different behavior. While pairwise functional connectivity of the SF population is significantly reduced at the transition from ACh^–^ to ACh^+^ (Fig 2C, left panel; *p* = 0, KS test), the EB neuron population becomes more strongly connected (Fig 2C, right panel; *p* < 4.75e-128, KS test).

Taken together, these results indicate that neurons with weak functional connectivity in NREM (in the sham/baseline condition *in vivo*, and the SF population *in silico*) undergo the large drop in functional connectivity during REM, while those with strong functional connectivity in NREM (in the post-CFC condition *in vivo,* and the EB population *in silico*) have a minimal drop or increase in connectivity. High co-firing in NREM (the ACh^–^ state) as a result of prior learning (modeled here as strengthened EB neuron synapses) enables the persistence of strong functional connectivity into REM (ACh^+^ state).

Further comparison of the experimental SHAM and CFC distributions (Fig 2B; left right panel) indicates that during exposure to CFC, there is rapid network reorganization leading to more heterogenous interactions between populations of putative excitatory neurons. This in turn leads to further selective strengthening of pairwise functional connectivity (i.e., recruitment) during REM – an effect observed to a much smaller extent during baseline and SHAM.

### ACh^–^ and ACh^+^ states differentially recruit a heterogenous population of SF neurons

**Fig 3:**
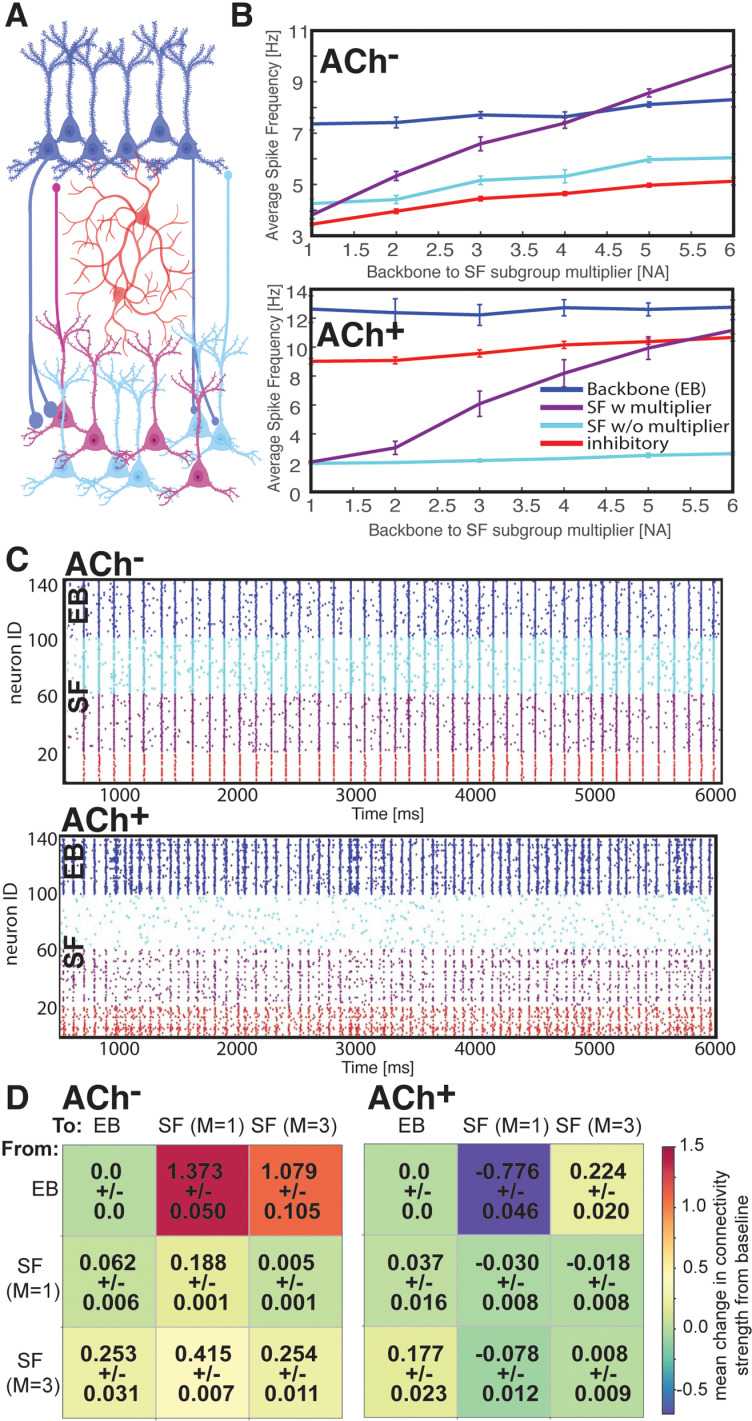
ACh^–^ and ACh ^+^ states differentially affect STDP-mediated recruitment of heterogenous populations of SF neurons into an engram. While ACh^–^ mediates broad recruitment of neurons into the engram, ACh^+^ drives selective recruitment of the more strongly connected population. ***A)*** Schematic of the model network, consisting of memory-encoding EB neurons (**EB**, ***dark blue***), inhibitory interneurons (***red***), and an SF group divided into two subpopulations - one receiving standard input from EB neurons as shown in Fig 1 (w/o multiplier; ***teal***) and one receiving variably enhanced input from the EB population (w multiplier; ***violet***). ***B)*** Average firing frequency during ACh^–^ (**top**) and ACh^+^ (**bottom**) for the four neuron populations, as a function of the SF subpopulation’s multiplier ($M_{ij}$) value. ***C)*** Representative raster plot of firing during ACh^–^ (**top**) and ACh^+^ (**bottom**) for the four neuron populations, using multiplier value of $M_{ij}=3$. ***D)*** STDP-driven connection strength changes between and within the excitatory populations (i.e., backbone neurons and the two SF populations), for ACh^–^ ($g_{Ks}=1.5\frac{mS}{{cm}^{2}}$, **left**) and ACh^+^ ($g_{Ks}=0\frac{mS}{{cm}^{2}}$, **right**). Plasticity between neurons within the backbone population is set to zero. Values indicate mean ± SEM across 4 simulation runs.

We next tested how the ACh^–^ and ACh^+^ state differentially recruit SF neuron populations into engrams, with varying degrees of excitatory input from the EB neurons (Fig 3). We divided the SF population into two groups - one receiving only weak synaptic input from EB neurons, and a second where EB neuronal input is strengthened (i.e., synapses originating from the EB to this SF population are multiplied by a factor $M_{ij}\in [1.0\;,\;6.0]$; Fig 3A). We monitored population responses of SF neurons as a function of this synapse multiplier $M_{ij}$ during the ACh^–^ and ACh^+^ states. In ACh^–^ conditions, as observed previously, the SF population was disinhibited due to decreased firing rates among inhibitory interneurons (Fig 3B, top, red line). This allowed both SF groups (those with and without the input multiplier) to fire in synchronous bursts with EB neurons (Fig 3C, top), leading to their recruitment into the engram via STDP-mediated strengthening of inputs from EB neurons [87] (Fig 3D, left). Fig 3D depicts changes in mean synaptic strengths between the three populations of excitatory cells (EB neurons, SF neurons with $M_{ij}=1$, and SF neurons with $M_{ij}=3$). During the ACh^–^ state, connections between EB neurons and both SF groups were strengthened; strengthening also occurred to a lesser extent at synapses between neurons in the SF groups. In contrast, during the ACh^+^ state, strong inhibitory input (Fig 3B, bottom, red line) suppressed the activity of those SF neurons with less excitatory input from EB neurons (i.e., only SF neurons with high $M_{ij}$ are active; Fig 3B, bottom, teal line). The SF neurons receiving enough excitatory input to remain active in the ACh^+^ state synchronized with the EB population in coherent bursts (Fig 3C, bottom). This bursting drove STDP-mediated strengthening of connections from the EB neurons to the active SF neurons. At the same time, noisy uncorrelated activity among the strongly inhibited SF neurons induced weakening of their synaptic connections (Fig 3D, right).

### ACh^–^ and ACh^+^ states play distinct roles when multiple engrams are consolidated simultaneously

**Fig 4:**
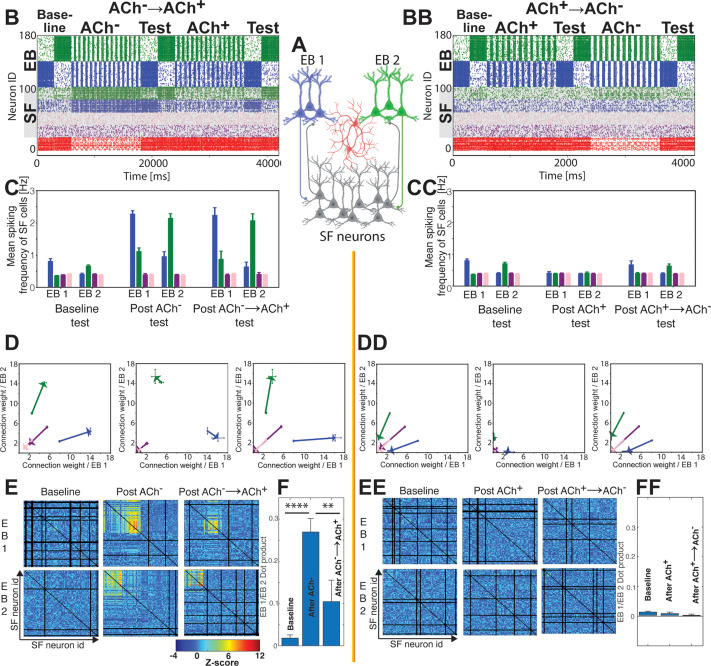
Differential roles of ACh^–^ and ACh^+^, and benefits of the ACh^–^→ACh^+^ sequence during consolidation of multiple memories. Network simulations are run with stereotypic ACh^–^ →ACh^+^ sleep architecture (**left column; *B-F***) or with reversed ACh^+^→ACh^–^ architecture (**right column; *BB-FF***). During stereotypic sleep architecture ACh^–^ mediates broad recruitment of SF neurons into engram representations, while subsequent ACh^+^ prunes the representations to keep them distinct. ***A)*** Schematic of the model network, where the EB population consists of two separate groups encoding EB1 (***blue***) and EB2 (***green***). ***B)*** Representative raster plot of network activity, where retrieval/activation patterns of SF neurons are tested in response to EB1 and EB2 backbone population activation before sleep (baseline), after ACh^–^, and after ACh^–^→ACh^+^. Tests occur in high ACh ($g_{Ks}=0.3\frac{mS}{{cm}^{2}}$), with continuous input from one of the two backbone populations, and no synaptic plasticity. Backbone neurons’ firing is not shaded (neuron IDs 100-180); firing of all SF neurons is shaded in gray (neuron IDs 20-80). SF neurons receiving stronger input (i.e., more connections at baseline) from the EB1 backbone population are shown in blue; those receiving stronger input (i.e., more connections at baseline) from the EB2 backbone population only are shown in green. Pink and violet SF neurons indicate populations receiving weak (i.e., least connections from both backbones at baseline) and strong input from both EB populations (i.e., most connections from both backbones at baseline), respectively; these groups receive lower constant current ($I_{drive}$) than green and blue SF groups, which leads to reduced, more random, firing patterns. ***C)*** Firing frequencies of SF population during three test phases (baseline, post-ACh^–^, and post-ACh^–^→ACh^+^). Colors depict groups as defined among the SF population in **B)**. ***D)*** Changes of synaptic connectivity between the two EB populations and the four SF groups, between following timepoint tests: baseline to post-ACh^–^ (left), post-ACh^–^ to post-ACh^+^ (center), and baseline to post-ACh^–^→ACh^+^ (right). X-axis denotes mean connection weight from EB 1 backbone to SF layer cells; the Y-axis denotes mean connection weight (bottom) from EB 2 backbone to SF layer cells. The vectors demark the change of the mean connection strengths between the afore mentioned timepoints (see S4 Fig) ***E)*** Functional connectivity between individual pairs of SF neurons during memory tests. Colors indicate the statistical significance (bootstrapped Z-score) of connections during baseline, post-ACh^–^ and post- ACh^–^→ACh^+^ tests for a representative simulation. ***F)*** Overlap of functional connectivity patterns among SF neurons during reactivation of EB 1 vs. EB 2 backbone populations, during baseline, post-ACh^–^ and post-ACh^–^→ACh^+^ tests. Values indicate mean ± SEM for 4 simulation runs; **** and ** indicate *p* = 0.004 for baseline vs. post-ACh^–^ and *p* = 0.033 for post-ACh^–^ vs. post-ACh^–^→ACh^+^, respectively. During reverse sleep architecture the recruitment of the new cells into representations is effectively abolished and consequently there is no memory consolidation. *BB - FF)* Panels showing functional connectivity changes (similar to ***B – F***) for a reversed-architecture sleep cycle (i.e., ACh^+^ followed by ACh^–^. ACh^+^→ACh^–^ state ordering leads to weakened connectivity between memory-encoding backbone neuron populations and SF neurons, leading to failed recruitment of SF neurons into memory traces during sleep.

Having characterized network and neuronal changes in the ACh^–^ and ACh^+^ states during consolidation, we next tested how ACh^–^ and ACh^+^ states affect STDP-mediated recruitment of SF neurons by EB populations for multiple (two) simultaneously encoded memories (Fig 4A). An additional 40 EB neurons were added to the network to comprise a second engram population (EB 2, green neurons, Fig 4A). Due to the limited size of our network, engram populations occupy a large fraction of the total number of cells and would be strongly directly connected to one another under completely random connectivity. Thus, connections between EB 1 and EB 2 were removed to prevent runaway excitation, and activity of one EB was always paired with injected current suppression of the other backbone, in an effort to mimic the temporally segregated offline reactivation of hippocampal ensembles corresponding to distinct waking experiences [88,89]. Connections from the EB populations to the SF neurons were random. Thus, the number of connections each SF neuron received from either EB1 or EB2 varied. For visualization purposes, we divided the SF population into four quartiles based on the number of connections to each EB (Fig 4B and 4BB): 1) those receiving many connections from EB 1 and few from EB 2 (blue with gray background), 2) those receiving many connections from EB2 and few from EB1 (green with gray background), 3) those receiving few connections from either EB 1 or EB2 (pink with gray background), and 4) those receiving many connections from both EB1 and EB2 (violet with gray background). To prevent chronic self-excitation and complete synchronous activity among the SF population, we applied a hyperpolarizing input ($I_{drive}=-6\frac{\mu A}{{cm}^{2}}$) to both the pink and violet SF groups (3 and 4 above) so that they fired randomly and sparsely and were not recruited into either engram. In addition, the blue and green SF groups (1 and 2 above) had their inputs from EB1 and EB2 initially strengthened 20%, respectively ($\omega_{ij}(t=0)=1.2;\;$ see Methods). We tested the degree to which this selective strengthening impacted our results (S3 Fig), and found that our main results were robust to the removal of this bias. We then utilized this two-engram setup to illustrate how sequential ACh^–^ and ACh^+^ states impact the integration of EB1 or EB2-biased SF neurons into either engram. We monitored this network-wide integration during “test” bouts, which very loosely represent wake-state memory recall. These test bouts had a simulated ACh concentration nearly as high as the ACh^+^ (REM-like) state ($g_{Ks}=0.1\frac{mS}{{cm}^{2}}$) and no synaptic plasticity. During testing, each of the two initial EB populations were selectively activated with constant intrinsic current, which serves as a proxy for external (e.g., sensory) input to the network during recall. To identify network-wide effects driven by NREM vs. REM-like conditions, these tests occurred at baseline (i.e., before the ACh^–^ state), after the ACh^–^ state, and finally after the ACh^+^ state (Fig 4B). Within the ACh^–^ and ACh^+^ states, STDP occurred for all excitatory synapses of SF neurons. We observed that during baseline testing, blue and green SF neuron populations fired only sparsely in response to activation of either EB1 or EB2. During the subsequent ACh^–^ state, a lower level of inhibition allowed both blue and green SF populations to be active, spiking in bursts triggered by either EB1 or EB2. This led to relatively broad STDP-mediated potentiation of connections between each EB population and both populations of SF neurons. This potentiation, in turn, led to increased and overlapping activation of the SF populations during post-ACh^–^ testing, where the blue and green SF populations were partially activated by either EB population, causing overlap between the two expanded engram representations. However, in the subsequent ACh^+^ state, greater network inhibition prevented most SF neurons receiving EB input from spiking. Only SF neurons with a sufficiently biased input from one of the EB populations underwent the necessary synaptic strengthening (i.e., in the preceding ACh^–^ state) to maintain high activity in the ACh^+^ state that followed. These ACh^+^ dynamics caused STDP-mediated pruning of connections between each EB population and most of the SF neurons in the network – with only the strongest connections being spared. Thus, during the subsequent post-ACh^+^ test, there was greater segregation of activity patterns between the blue and green SF populations – with SF neurons activated by EB1 vs. EB2 becoming largely non-overlapping, and resulting in two expanded, but separated, engram populations.

To quantify recruitment of SF neurons into each engram, we characterized changes to three observables during the three test bouts:1) the firing frequency of the four SF neuron groups (Fig 4C); 2) changes in connection weights between EB1 and EB2 populations and the SF neuron groups (Figs 4D and S4), and 3) changes in patterns of pairwise functional connectivity among the SF neurons (Fig 4E and 4F). The firing rate of SF neurons coactivated with EB populations during the ACh^–^ state increased substantially from the baseline test to the post-ACh^–^ test. This was true for both the weakly coupled group pairs (green SF driven by EB1, blue SF driven by EB2), and between the preferentially coupled group pairs (blue SF driven by EB1, green SF driven by EB2; Fig 4C, p < 0.001, two sample t-test for all comparisons). The subsequent ACh^+^ state raised some SF neuron firing rates and lowered others as measured in the post-ACh^+^ test (Fig 4C, post-ACh^–^ vs. post-ACh^+^). However, there was no significant change in mean spike frequency after the ACh^+^ state, compared to mean firing rates at testing after the ACh^–^ state (*p* > 0.21, two sample t-test; Fig 4B) S4 Fig (diagonal) depicts sample distributions of all connection strengths from EB1 and EB2 to the SF neuron subgroups during each test bout. The mean change in connection strength between test bouts (averaged over four simulation runs) is depicted in Fig 4D and the off-diagonal terms of S4 Fig, with colored vectors corresponding to changes in mean excitatory connection weight incurred during the ACh^–^ state (Fig 4D, left panel;), the ACh^+^ state (center panel) and as a result of both sequential states (right panel). Synaptic changes reflected the changes seen in firing frequency – the ACh^–^ state caused general synaptic strengthening, with preference for the more densely connected SF group, and ACh^+^ caused selective pruning of synapses, where weaker connections (EB1 to green SF, EB2 to blue SF) were further weakened and stronger connections (EB1 to blue SF, EB2 to green SF) were more variably altered (S4 Fig, compare post-ACh^–^ and post-ACh^+^). Thus, the ACh^–^ state induces strong potentiation to the engrams, and the following ACh^+^ state depresses some synapses and strengthens others. Ultimately, the ACh^–^→ACh^+^ sequence resulted in synaptic changes recruiting two separate populations of SF neurons, one to each of the two EB populations (the effects of which are visible in Fig 4E and 4F).

This effect is further illustrated in the coactivation patterns of the EB and SF populations. We analyzed patterns of functional connectivity within the SF neuron population, using the functional connectivity algorithm described for Fig 1E [81]. We used this metric to characterize pairwise coactivation patterns between SF neurons when EB1 and EB2 were activated separately during each test (Fig 4E). While no significant functional connectivity between individual SF neuron pairs was observed during the baseline test, strong functional connectivity was observed both between and within green and blue SF neuron groups after the ACh^–^ state. After the ACh^–^ →ACh^+^ sequence, functional connectivity between the two SF groups was reduced significantly, but intra-group connectivity was preserved. To further address this, we quantified the degree of overlap between the portion of these matrices corresponding to the blue and green SF neuron populations (upper left quadrant) by computing the dot product between test pairs (Fig 4F). The functional connectivity pattern overlap is zero during the baseline test, increases significantly after the ACh^–^ state, and decreases significantly after the ACh^+^ state. We also measured the network-wide distribution of pairwise functional connectivity among all SF neurons (S5 Fig) during test-associated activation of either EB1 (S5A Fig, top) or EB2 (S5A Fig, bottom). After the ACh^–^ state alone, a large portion of the SF population exhibited increased functional connectivity, skewing the distribution towards positive values (S5A Fig, left panels). In comparison, after the following ACh^+^ state, there was a strong tendency for functional connections to be weaker, although a small fraction of pairs had increased connectivity compared to the post-ACh^–^ test bout (S5A Fig, center panels). Thus, while synaptic weakening and loss of functional connectivity was the predominant effect of the ACh^+^ state, some functional connections became stronger during this time. Lastly, we compared changes in the functional connectivity between the baseline test and the post-ACh^–^→ACh^+^ test S5A Fig, right panels). While most functional connections among the SF population were not significantly changed, a small number were increased, corresponding to SF neurons that were selectively recruited into either of the two EB representations (Fig 4E). These results demonstrate the potential importance of a low ACh state followed by a high-ACh state in expanding coactive groups of cells in a manner that maintains separation between which new cells become functionally connected to which engram.

To test the robustness of these findings, the duration of depolarizing current pulses used for reactivation of the two EB populations during the ACh^–^ and ACh^+^ states, as well as the temporal separation of reactivation for the two populations, were systematically varied (S6 and S7 Figs). The duration of reactivation had little impact on either the normalized firing rate of the blue/green SF neurons (referred to as “activation”, see Methods), or the normalized difference in firing rate between the two SF populations (referred to as “segregation”, see Methods – value of one indicates total segregation S6 Fig). On the other hand, the temporal separation of reactivation had an impact on both properties, with longer delays between reactivation of EB1 and EB2 populations leading to decreases in both metrics. However, lengthening the total duration of reactivation (i.e., increasing the total number of reactivations) could compensate for the effects of longer delays on both engram activation and segregation (S7 Fig).

We find that there are network conditions which could interfere with these mechanisms. Our data suggest that the dynamics of the ACh^–^ state induce somewhat indiscriminate synaptic potentiation between EB neurons and the recruitable SF population. The resulting population of SF neurons with large numbers of connections from both EB populations, and potentially a high degree of excitability, could make engram segregation in the subsequent ACh^+^ state difficult. To investigate this, we ran a series of simulations suppressing the violet SF population (which initially receives strong input from both EB populations) to various degrees. We varied the difference in the $I_{Drive}$ between the violet SF population and the blue and green populations (S8 Fig). Not surprisingly, we observed that with strong activation of the violet population, the segregation of the blue and green SF groups is reduced significantly, whereas the activation of the blue and green groups remains high, with both populations being driven by elevated violet SF spiking (S8 Fig). This suggests that in networks with insufficient heterogeneity, such as highly recurrent networks, those with a small variance in the number of excitatory inputs per neuron, or those within highly overlapping engrams, maintaining separate representations during consolidation will be challenging.

Together, these data suggest that during ACh^–^→ACh^+^ sequences, multiple engrams are able to recruit additional network neurons, and can do so in a manner that preserves the separation of their neural representations. These results suggest that the sequential changes in cholinergic tone across the canonical NREM→REM cycle may drive the recruitment of distinct neuronal populations into existing memory engrams, providing a mechanism for the simultaneous consolidation of multiple, distinct memories in a single network.

### Sequential ACh^–^→ACh^+^ state ordering is essential for selective engram formation

Because wake→NREM→REM sleep state sequences are ubiquitous across animal species (and wake→REM→NREM sequences are not typically observed), we were curious how reversing the sequence would impact the consolidation process. We tested the effects of the ACh^+^ state preceding the ACh^–^ state (Fig 4BB-FF; right column). In this scenario, we observed diametrically different SF population dynamics (Fig 4BB). Increased synaptic inhibition during the ACh^+^ state led to suppressed activity among SF neurons across the entire simulation (Fig 4CC). Synaptic inputs from EB populations, once weakened during the suppressive ACh^+^ state, could no longer drive burst firing among SF neurons during the subsequent ACh^–^ state. This eliminated recruitment of the SF neuron population into engrams during the ACh^–^ state. This effect was evident as weakened EB input connections to SF neurons after the ACh^–^ state (Fig 4DD), effectively disconnecting SF neurons from the EB populations. Functional connectivity clusters did not form in this scenario (Fig 4EE) and the overlap between EB1 and EB2 functional connectivity remained at zero (Fig 4FF), indicating a complete lack of recruitment of SF neurons into the two engrams. There were also no observable changes in the functional connectivity distributions across the network (S5B Fig). Thus, if the typical ACh^–^→ACh^+^ sequence (corresponding to NREM→REM) is reversed to ACh^+^→ACh^–^ (REM→NREM), the result is a lack of engram enlargement – interpretable as a failure of memory consolidation.

### Multiple ACh^–^→ACh^+^ sequences drive optimal segregated engram enlargement

**Fig 5:**
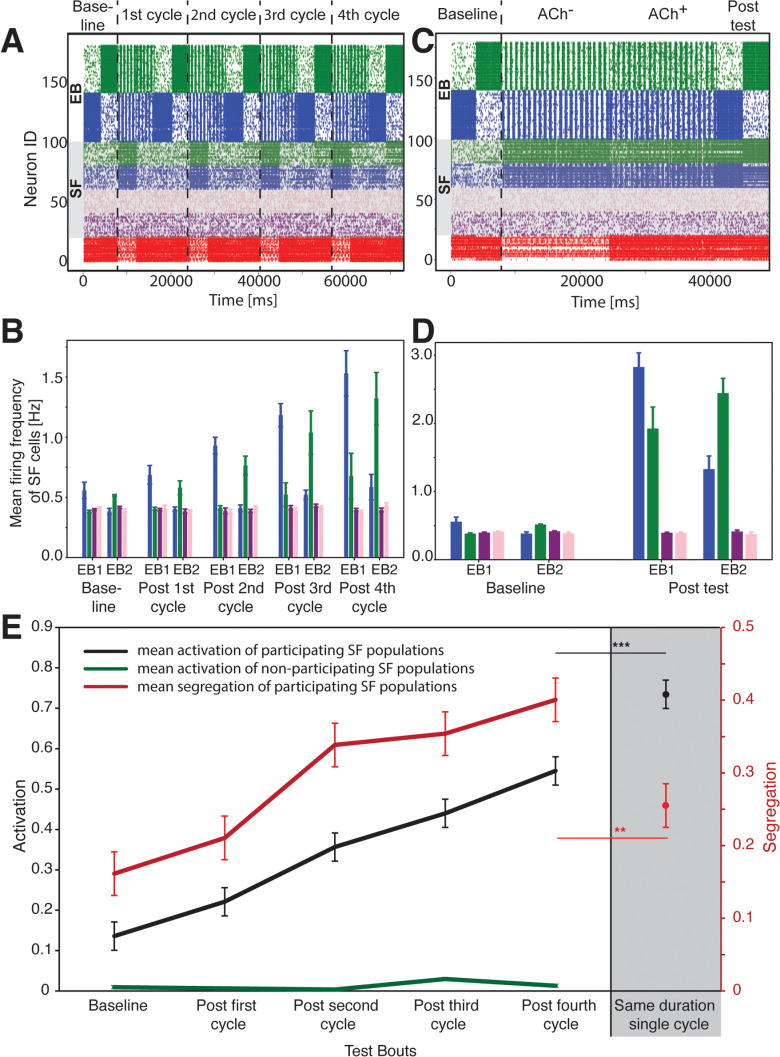
Benefits for consolidation of multiple ACh^–^→ACh^+^ cycles. Comparison of activation and segregation of two memories when sleep consists of 4 repeating ACh^–^→ACh^+^ cycles, with the case when there is only one cycle present of the same cumulative duration. **A)** Representative raster plot over four ACh^–^→ACh^+^ cycles. Memory retrieval tests were performed as described for Fig 3, after every ACh^–^→ACh^+^ sequence. **B)** Mean firing responses within SF groups during activation of EB 1 or EB 2 backbone neuron (EB) populations. **C)** Representative raster plot depicting a single, extended ACh^–^→ACh^+^ cycle (i.e., of the same total duration as the four cycles in **A**). Retrieval tests were performed before and after the ACh^–^→ACh^+^ sequence. **D)** firing responses within SF groups during retrieval-associated activation of EB 1 or EB 2 backbone neuron populations. **E)** Changes in activity profiles (activation and segregation; see **Methods**) for SF groups after multiple cycles vs. a single sleep cycle of the same total duration (**gray area**). Multiple sleep cycles result in greater segregation of engram representations corresponding to different memories. While activation of SF neurons increases for both cases (i.e., four repeating ACh^–^→ACh^+^ cycles as well as one sleep cycle), indicating their broad recruitment into the engram representations, the segregation for a single sleep cycle does not significantly increase from baseline levels suggesting coactivation of recruited cell populations and thus high overlap of the representations. Thus, iterative ACh^–^→ACh^+^ cycles mediate balance between recruitment of additional cells into an engram and the same time separability of independent representations. Values indicate mean ± SEM for 4 simulation runs; *** and ** indicate *p* = 0.004 and *p* = 0.038, respectively, for activation and segregation.

Sleep, across species, occurs in repeating cycles of NREM and subsequent REM. These cycles occur either intermittently throughout the day, or in the case of humans and some other species, in succession throughout a longer, consolidated sleep period. An unanswered question is whether, and why, it might be beneficial to have multiple NREM→REM cycles, rather than a single, massed period of NREM and REM. To address this, we ran two sets of simulations. In the first, we divided the sleep period into four repeating ACh^–^→ACh^+^ cycles, with each cycle followed by a test in which EB1 and EB2 were separately activated as done previously (Fig 5A and 5B). In the second set of simulations, a single test was run after one long ACh^–^→ACh^+^ sequence, with the total duration of ACh^–^ and ACh^+^ states matched to the four cycle simulation (Fig 5C and 5D). For the tests interspersed between the four shorter cycles (Fig 5B), the green and blue SF neuron groups activated preferentially with their respective EB population, while maintaining separation. In contrast, after the longer-duration, single NREM→REM cycle, there was significant co-activation of these SF neuron populations during the final test (Fig 5D). We applied the activation and segregation metrics to these simulation sets (Fig 5E, see Methods), and observed both activation and segregation increased steadily as a function of the number ACh^–^→ACh^+^ cycles. In contrast, the activation for the non-participating SF neuron groups (pink and violet) did not change throughout the cycles (green line). The outcome of a longer-duration, single ACh^–^→ACh^+^ cycle was qualitatively and quantitatively different from the four-cycle case. Activation of the blue and green SF neuron groups was significantly greater than after cycling ACh^–^→ACh^+^ states, but their segregation was decreased. This means that while these SF groups were more active with EB1 and EB2 after a single, long cycle compared to multiple short ACh^–^→ACh^+^ cycles, the overlap in activity across reactivation of both backbones was much higher. This is because during a single extended period of the ACh^–^ state, synaptic connections from the backbone populations to green and blue SF neuron groups underwent continuous strengthening, as well as the interconnections between the two SF groups. Once these connections became sufficiently strong, the subsequent ACh^+^ state was unable to silence most of the blue/green SF neurons. Activity in the ACh^+^ state became consistent co-activation of these populations, further strengthening most synapses and avoiding pruning. Taken together, these data suggest that under certain circumstances (e.g., when consolidating more than one memory in a network simultaneously), multiple NREM→REM cycles can be more effective than a single, longer-duration cycle. In other words, multiple NREM→REM iterations are most optimal for keeping engrams segregated.

## Discussion

Vertebrate sleep cycles, while variable in length, almost invariably follow the NREM→REM order [90]. However, the precise roles of NREM and REM in sleep functions such as memory consolidation [2,62], and the functional significance of this ubiquitous sleep state ordering, are unknown. Sleep has been proposed to play various roles in regulating synaptic changes in the context of memory consolidation [3], from reactivation of neurons and networks engaged by prior learning [21,89,90], to regulation of excitatory-inhibitory balance [8,13,91], to homeostatic downscaling of synapses [92,93].

Here we defined two discrete and distinct populations: a highly excitable engram population that drives reactivation during sleep, and a slow firing, plastic population that can be recruited into the engram during sleep consolidation. We concentrate here on the dynamics of this second group, however there is no doubt that neurons in the first group could also remap their firing patterns as the connectivity of the engram evolves during consolidation. In this context, we have defined a simplified biophysical model in which network dynamics are regulated through changes in a muscarinic M1 ACh receptor-modulated current. We show that cholinergic modulation alone, during NREM and REM sleep, may drive these states to play distinct and complementary roles in sleep-dependent memory consolidation. We show that changing ACh level directly modulates neuronal excitability via this pathway, and that this in turn affects the excitatory/inhibitory balance of the network. During the ACh^–^ state, low ACh lowers the activity of inhibitory interneurons, leading to network disinhibition. This scenario allows for STDP-driven recruitment of initially slow-firing excitatory neurons into memory engrams, driven by engram backbone populations (Figs 1 and 3). In contrast, during the ACh^+^ state, the M-current is suppressed by high ACh levels, and the resulting elevated network inhibition (Fig 1) suppresses activity in most of the slow-firing excitatory neurons (sparing only those most strongly driven by backbone populations), resulting in the competitive pruning of neurons newly recruited into memory engrams (**Fig 3**). This mechanism captures changes in firing statistics observed experimentally in CA1 in the hours following CFC in mice (as opposed to sham situation, Fig 2), where REM generally exhibits lower functional connectivity across excitatory neurons as compared to NREM, except for a limited population of cells that have their functional interactions strengthened.We further show that ACh^–^ →ACh^+^ sequential cycling plays an especially critical role when consolidating multiple memories (Fig 4), as it allows for engram expansion while keeping the representations of consolidating memories separated from one another. Within the model, reversing the sequence (i.e., ACh^+^→ACh^–^ leads to a failure of engram recruitment (Fig 4) and replacing multiple ACh^–^ →ACh^+^ cycles with one prolonged sleep cycle leads to a failure of engram segregation when two engrams are being consolidated simultaneously (Fig 5). Together, these findings provide a theoretical basis for why sleep architecture and transitions may be critical for the memory-processing aspects of sleep function.

While there is currently no direct experimental evidence that the neuromodulatory effects of ACh mediate specific memory-related functional roles for NREM and REM sleep as described here, there are many lines of evidence to indirectly support this concept.

### Engram formation and maturation

Available data suggest that engram populations are formed rapidly in the context of waking learning experiences [94,95]. The engram neurons themselves are more excitable and form a strongly interconnected population [66,67,96–100]. The engram remains highly dynamic and undergoes maturation during consolidation, and is hypothesized to further expand (recruiting additional neurons into the memory representation) and/or strengthen connections within the memory trace [64,69], while at the same time increasing separation from other memory-encoding neurons, to allow for accurate recall in the future. It is established that during post-learning sleep, these engram neurons preferentially fire in association with ripples, show increased bursting and temporal coactivation [65], and selective place field remapping [59,101].

### Differential roles of NREM and REM during memory consolidation

Behavioral studies in both human subjects and animal models have aimed to identify precise roles of NREM vs. REM sleep in specific aspects of memory consolidation, to whether the two states have differential roles. The importance of NREM sleep for consolidating many forms of memory (including declarative and procedural memory) has been well established, based on studies either disrupting NREM behaviorally, targeting state-specific features of NREM, or enhancing NREM-specific network activity patterns [13,15,17,22,102].

Effects of REM sleep-targeted deprivation studies in human subjects have been less clear, with many forms of memory consolidation (particularly declarative memory) proving robust to this manipulation. However, some sleep-dependent forms of consolidation are disrupted by prevention of REM, including studies where multiple associations (e.g., of different visual cues with an aversive shock vs. safety) must be made in more complex scenarios [103]. A converse approach, which has been used successfully to address REM’s cognitive functions in human subjects, is identification features of behavioral tasks where benefits are correlated with post-learning REM sleep. In one such study, participants were trained on two competing sensory discrimination tasks in rapid succession, followed by a nap opportunity. Only those who engaged in a post-learning nap containing REM showed perceptual improvement for both tasks, without interference between them [61]. Finally, a recent study used administration of ACh receptor antagonists to block ACh transmission during post-learning REM sleep in human subjects, and found subtle deficits in consolidation. Intriguingly, however, subjects in the study were trained on two separate memory tasks prior to the intervention [104]. Together, these data support the idea that REM sleep with high ACh signaling could provide an opportunity to segregate competing memories from one another, to improve performance on complex tasks and/or prevent interference between memories.

At the same time, it has been shown targeted memory reactivation (i.e., via sensory cueing of material learned prior to sleep) seems to ‘work’ only in NREM sleep and not REM [105]. Our modeling predicts that targeted memory reactivation (driving EB neuron reactivation in our model) would have less of an effect during REM due to the increased inhibition in the network.

REM also seems to facilitate other cognitive functions, which may not immediately appear related to a role in memory storage. Intriguingly, data from human subjects indicates that following a period of REM (but not NREM), participants show increased creative thinking, have greater ability to form new associations, and are more likely to have restructured learned information [106,107]. These features are not incompatible with the “pruning” aspects of REM described here, insofar as elimination of weak or irrelevant connections throughout a network may increase its capacity for forming new connections upon receiving new inputs. Experimental results from both humans and animals show that the elevated neural inhibition observed during REM protects memories from interference to permit continual learning [108].

Recent experimental data [109,110] indicate that memories carrying emotional silence specifically benefit from the integration of REM into sleep cycles, with NREM sleep consolidating memory while REM filters memory for continued processing (i.e., further consolidation) based on the saliency (e.g., emotional tone) of the memory. Our modeling is consistent with these results. Representations are pruned during the ACh^+^ state, leaving only the strongest excitatory connections, because of increased inhibition. This competitive environment could lead to reactivation of only more salient memories and competitively disadvantage weaker memories. To illustrate this effect, we ran simulations (S9 Fig) in which one EB population was reactivated for a comparatively longer period (S9A Fig) or with less excitatory drive (S9B Fig), than the other. We show that the EB populations with less or weaker reactivation have reduced neuron recruitment in the ACh^–^ state and increased pruning in the ACh^+^, state resulting in less SF co-activation during test periods. Based on these and other results, theories of consolidation involving both sleep stages are gaining support, positing roles for NREM and REM aligned to our modeling results [111,112].

### Roles of cholinergic signaling in regulating network dynamics during memory consolidation

It is known that both principal cells and subpopulations of inhibitory interneurons express the M1 receptor, with especially prominent expression in the hippocampus and neocortex [39,48,113,114]. Altering state-specific ACh and/or M1-mediated signaling impairs consolidation [56,57,115–117]. For example, preventing the normal decrease in ACh signaling during NREM sleep disrupts memory consolidation, in both human subjects [54] and mice [13].

ACh preferentially activates interneuron populations in the hippocampus [49,50] and neocortex [118–120]; this selective sensitivity is mediated by both nicotinic and muscarinic receptors [51,121]. Because ACh release from medial septal inputs to hippocampus, and by basal forebrain inputs to neocortex, is higher during wake and REM than NREM sleep [122–125], this indicates that, as is true in our model, these networks may be gated by interneurons during REM and disinhibited during NREM. Recent findings also support the idea that altered inhibitory signaling may expand memory traces during post-learning sleep. In regions such as the dentate gyrus, suppression of inhibitory transmission during sleep seems to be an essential component of memory processing [13]. Available data suggests that reactivation and replay of memory traces occurs preferentially (and more broadly throughout the network) during NREM as a correlate of active memory consolidation [15,16]. Finally, there is accumulating evidence of synaptic strengthening occurring preferentially during post-learning NREM sleep. The analyses shown in Fig 2 illustrate that functional connectivity between CA1 neurons is stronger during post-learning NREM than post-learning REM, except for a smaller number of pairwise interactions that show competitive strengthening. *In vivo* electrophysiological recordings have demonstrated baseline [18,101] or learning-driven [17,19] firing rate increases that occur selectively across NREM bouts. *In vivo* recordings have shown that potentiation of thalamocortical synapses occurs selectively during NREM bouts [126]. Finally, *in vivo* imaging data have illustrated dendritic spine formation in the neocortex during post-learning NREM sleep [10].

There are also strong data to support the notion that REM facilitates inhibition-driven pruning of memory traces [111]. Recent *in vivo* imaging data have shown that in neocortex, interneuron-mediated suppression of pyramidal cell activity is higher during REM sleep compared with NREM sleep [8]. The idea that excitatory synapses are selectively pruned during REM (but not NREM) is supported by *in vivo* electrophysiological recordings from neocortex and hippocampus (where principal cell firing rates decline selectively across REM bouts) [101,127] and *in vivo* imaging studies of neocortex (where dendritic spines are lost preferentially during REM) [9]. More generally, recurrent feedback inhibition (which our modeling suggests is higher in REM) is thought to promote competition amongst principal neurons throughout the brain. Reliable spiking of interneurons is observed in the hippocampus and other structures after spontaneous pyramidal cell firing [128], or after optogenetic activation of one to several pyramidal cells [129–131]. On the other hand, optogenetic silencing of principal neurons can lead to strong disinhibition of neighboring principal cells [132,133]. This mechanism is known to be important in regions like the dentate gyrus, where interneuron populations are known to gate the population of principal neurons activated during memory consolidation [13] and recall [133]. It has also been shown that inhibition may play an important role in sculpting the activation patterns of excitatory neurons during sharp-wave ripples [134–136].

## Limitations of the study

This study investigates the possible role of muscarinic M1 receptor-mediated modulation of network activity during NREM and REM sleep on sleep-dependent memory consolidation. This receptor, while being the most abundantly expressed cholinergic receptor in hippocampus and neocortex, is only one of several other muscarinic and nicotinic acetylcholine receptor types, which are not explored in this model. ACh concentration also fluctuates within NREM and REM, making these states more dynamic than we account for in our model. Further, our study considers a simplified, reciprocally connected excitatory/inhibitory network which has some similar characteristics to the hippocampal region CA3, but does not attempt to recreate any specific brain circuit at a detailed level. Thus, only general conclusions can be drawn based on our model on the role of M1 receptors in producing the network dynamics observed, and the potential effects of those dynamics during NREM and REM sleep on memory consolidation.

It is known that ACh inhibits excitatory and inhibitory synaptic transmission in the hippocampus [39,137,138]. We do not incorporate these effects into our model, as they are primarily important in modulating the degree to which hippocampal circuits are driven by external input (e.g., from entorhinal cortex), which falls outside the scope of our model. In addition, we expect that including this reduction in synaptic signaling during the high-ACh state in our model would mainly have the effect of recruiting a smaller subset of the most strongly potentiated SF cells to coactivate with the engram cells, likely enhancing the synaptic pruning effect of the high-ACh state.

Further, our model does not include the effects of noradrenaline and/or other neuromodulators [25]. Noradrenaline is low in both NREM and REM, but it has also been shown that its fluctuations underlie sleep oscillations patterns on a slow scale (0.2Hz) and coincide with spindle formation [139]. Sleep slow oscillations and spindles have been linked to memory consolidation [111], which themselves vary throughout NREM sleep. The interaction between slow oscillations, spindles, and sharp wave ripples linking the hippocampus and various cortical areas is posited to underlie their relevance to consolidation [140], however interaction between multiple brain regions is outside the scope of our current set of modelling results. As such, there are multiple consolidation-relevant aspects of sleep that our model does not account for.

For simplicity, the interneuron population in our model is uniform, incorporating properties of both SST and PV interneurons. There is exhausting evidence that inhibitory interneuron populations are very diverse (in terms of firing patterns and postsynaptic targets) in the hippocampus and in the cortex [48]. While we have modeled the mean inhibitory dynamics under the influence of ACh in these studies, current findings support the behavior of interneurons observed in our model. The general effect of ACh in both neocortical and hippocampal circuits is increased inhibition through activation of the interneuron population [13,138,141,142].

Sleep behavior, and proportions of NREM and REM sleep, change across the lifespan of most mammalian species. Our model does not address the potential differential effects of sleep in early life. It has been proposed that sleep’s role in nervous system function may transition in humans at about 2–3 years of age (from being used primarily for neural reorganization to being utilized for repair and clearance) [143]. Our current modeling and experimental data only address the role of sleep in memory consolidation, which is at least one of the critical functions of sleep in the adult brain [111]. Future experimental and modeling work may address how developmental stage-specific sleep patterns affect plasticity in neural circuits, and whether the underlying mechanisms mimic the effects of ACh^–^ and ACh^+^ states described here.

## Conclusions

In all, our present data provide a set of testable hypotheses about the differential roles that NREM and REM sleep play in sleep-dependent memory consolidation. They suggest specific neural network level mechanisms associated with NREM and REM in the consolidation process, wherein NREM drives expansion of memory traces and subsequent REM maintains optimal segregation of traces when multiple memories are being consolidated. The predictions indicate a critical role of REM-based pattern separation during storage of multiple correlated memories in terms of time and content. The observed reduction of overlap between the representations during REM maybe also important for preventing catastrophic forgetting (i.e., overwriting old memories with new ones) [144]. Finally, our data suggest an essential mnemonic function for the seemingly ubiquitous wake→NREM→REM ordering phenomenon, present in nearly all vertebrate species.

## Methods

### Ethics statement

All animal husbandry and experimental procedures were approved by the University of Michigan Institutional Animal Care and Use Committee (IACUC); protocol approval number: PRO00011982.

#### Neuron model.

Excitatory and inhibitory neurons were modeled using a conductance-based Hodgkin-Huxley formalism [145,146]*.* The time-dependent voltage $V_{i}$ of an individual neuron is given by the master equation:


$$C\frac{dV}{dt}=-g_{Na}m_{\infty }^{3}(V)h(V)\left(V-V_{Na}\right)-g_{Kdr}n^{4}(V)\left(V-V_{K}\right)-g_{L}\left(V-V_{L}\right){-g}_{Ks}z(V)\left(V-V_{K}\right)+I_{drive}+I_{noise}-I_{syn}$$ (1)

with the first four currents on the right-hand side representing a fast inward Na^+^ current, a delayed rectifier K^+^ current, a membrane leak current, and a slow outward (hyperpolarizing) K^+^ current representative of muscarinic M_1_ receptor activation (M-current). The M-current conductance, $g_{Ks}$, serves as a proxy for the state-dependent ACh level, with ACh reducing activation of this current. During NREM sleep, when ACh levels are low, M-current is high ($g_{Ks}=1.5\frac{mS}{cm^{2}}$); conversely, during REM sleep, when ACh concentration is high, M-current is blocked ($g_{Ks}\to 0$). On a cellular level, $g_{Ks}$ conductance modulates membrane excitability, with low $g_{Ks}$ (high ACh) yielding Type 1 excitability, whereas high $g_{Ks}$ (low ACh) yielding Type 2 excitability. Type 1 excitability is characterized by low firing frequencies in the absence of excitatory input, but a high frequency gain in response to input (i.e., a steep current-frequency [i-f] curve), and advance-only firing phase responses to brief stimulation pulse (i.e., phase response curve [PRC]). Type 2 excitability has a threshold in firing frequency onset, a shallow frequency gain function (i-f curve), and a biphasic PRC[149]. The parameters in the above equation (except for driving current [$I_{drive}$], described below) are the same for both excitatory and inhibitory interneurons, and are provided in Table 1.

**Table 1:** Cellular parameters of modeled excitatory and inhibitory neurons.

Parameter	Value
C	$1\frac{\mu F}{cm^{2}}$
$g_{Na}$	24 $\frac{mS}{cm^{2}}$
$g_{Kdr}$	$3\frac{mS}{cm^{2}}$
$g_{Ks}$	$0-1.5\frac{mS}{cm^{2}}$
$g_{L}$	$0.02\frac{mS}{cm^{2}}$
$V_{Na}$	55 mV
$V_{K}$	-90 mV
$V_{L}$	-60 mV

The gating variables, i.e., sodium inactivation, h(V), potassium activation, n(V), and M-current activation, z(V), are given by:


$$\frac{dh}{dt}=\left(h_{\infty }(V)-h\right)\frac{1}{\tau_{h}(V)}$$ (2)

$$\frac{dn}{dt}=\left(n_{\infty }(V)-n\right)\frac{1}{\tau_{n}(V)}$$ (3)

$$\frac{dz}{dt}=\left(z_{\infty }(V)-z\right)\frac{1}{75}$$ (4)

where ${\;h}_{\infty }(V)$, $n_{\infty }(V)$, $z_{\infty }(V)$, $m_{\infty }(V)$, $\tau_{h}(V)$ and $\tau_{n}(V)$ are defined as:


$$h_{\infty }(V)=\frac{1}{1+e^{\frac{V+53}{7}}},$$ (5)

$$n_{\infty }(V)=\frac{1}{1+e^{\frac{-V-30}{10}}},$$ (6)

$$z_{\infty }(V)=\frac{1}{1+e^{\frac{-V-39}{5}}},$$ (7)

$$m_{\infty }(V)=\frac{1}{1+e^{\frac{-V-30}{9.5}}},$$ (8)

$$\tau_{h}(V)=0.37+\frac{2.78}{1+e^{\frac{V+40.5}{6}}},$$ (9)

$$\tau_{n}(V)=0.37+\frac{1.85}{1+e^{\frac{V+27}{15}}},$$ (10)

where the time constants are expressed in [ms] and voltages in [mV]. External driving current, $I_{drive},$ is a parameter used to set the excitability of neurons. For excitatory neurons, $I_{drive}=0.5\frac{\mu A}{cm^{2}}$ unless otherwise specified, while for inhibitory neurons $I_{drive}=-0.1\frac{\mu A}{cm^{2}}$. The difference in excitability between the two populations ensures that inhibitory neurons fire only in response to excitatory input, providing natural competition between excitatory populations. Random input, $I_{noise}$, representing input from other external modalities, is modelled by a low-probability excitatory current influx to each neuron. Every millisecond there is a 0.002% chance that a given neuron will receive an $I_{drive}=80\frac{\mu A}{cm^{2}}$ input with duration of 1 ms. This results in spontaneous firing with mean frequency of 2 Hz for all neurons in the network. Finally, $I_{syn}$ represents synaptic input from other network neurons, and is described in detail below.

#### Network architecture.

***Single-memory network:*** The network encoding a single memory trace (Figs 1-3) is composed of 120 excitatory neurons and 20 inhibitory neurons (however we investigated consistency of our results for larger networks (3x), see supplementary data S1A Fig). Excitatory neurons provide sparse and random input to the network, with 10% connectivity to both excitatory and inhibitory neurons. Inhibitory neurons provide input to 50% of neighboring excitatory and inhibitory neurons within a specified connectivity radius. Excitatory neurons are divided into two groups, the encoded memory’s initial backbone (engram backbone EB) population (40 neurons) and less activated (SF) neurons (80 neurons). Backbone neurons represent the initial memory trace encoded during learning, receiving less inhibition than SF neurons, and having higher-efficacy, fixed synaptic weights between one another. SF neurons fire less due to decreased interconnectivity and increased inhibition (see section below). Excitatory synaptic inputs targeting SF neurons (i.e., from both engram backbone and SF neurons) undergo spike timing-dependent plasticity as described below.

***Two-memory network***: The two-memory network (Figs 4 and 5) is composed of 160 excitatory and 20 inhibitory neurons (however we investigated consistency of our results for larger networks (3x), see supplementary data S1B Fig). The difference in number of excitatory neurons stems from creating a second engram backbone, as these neurons are divided evenly into three groups – 80 SF neurons, 40 backbone neurons corresponding to EB 1, and 40 neurons corresponding to EB 2. The two memory-encoding backbone populations have no synaptic connections with one another, but provide random, sparse input to both inhibitory and SF neuron populations. Note that the two engram backbones are activated sequentially, thus the maximal current generated by EB population remains largely the same. Other two-memory network parameters are identical to the single-memory network.

Since input connections from each backbone population to SF neurons are random, the number of connections each SF neuron receives from either backbone population varies. We divided the SF neuron population into four quartiles (see S4 Fig): [1] stronger input from EB 1 (i.e., neurons receiving most connections at baseline from mem 1 backbone; denoted as blue in the SF population – gray background on Figs 4 and 5) – this quartile receives relatively strong input from the EB 1 backbone population and relatively weak input from EB 2, [2] **stronger input from EB 2 (i.e., neurons receiving most connections at baseline from mem 2 backbone; denoted as green in the SF population – gray background)** - this quartile receives relatively strong input from the EB 2 backbone population and relatively weak input from EB 1, [3] **weak input from both of the memories (i.e., neurons receiving least connections at baseline from both backbones; denoted as pink in the SF population)**, and [4] **strong input from both of the memories (i.e., neurons receiving most connections at baseline from both backbones; denoted as violet in the SF population)**. In most of the simulations, the last two populations (i.e., pink and violet) have sparse and random firing (driven by noise), as they receive lower $I_{drive}$ as compared to other SF groups. We have tested the effects of activating the population of SF cells receiving the largest number of synapses from both memory backbones (violet group), and as predicted we observe that strong activation of these neurons hampers segregation of the two memories (S6 Fig)

#### Synaptic communication and plasticity.

When each modeled neuron’s membrane voltage exceeds preset threshold (5 mV), an action potential is recorded, and the presynaptic neuron activates inhibitory or excitatory postsynaptic current to all its postsynaptic targets. The postsynaptic currents, $I_{syn}^{i}(t)$, received by an (i-th) cell are modeled as a linear combination of fast and slow excitatory and inhibitory currents.


$$I_{syn}^{i}(t)=\sum_{j}^{N_{e}}\sum_{k}A_{ij}{M_{ij}w}_{ij}^{E}(t)\left(V_{i}-E_{rev}^{E}\right)R^{E}\exp\left(-\frac{t-t_{jk}}{\tau_{f}}\right)+\sum_{l}^{N_{i}}\sum_{m}A_{il}M_{il}\left(V_{i}-E_{rev}^{I}\right)\left(R_{f}^{I}\exp\left(-\frac{t-t_{lm}}{\tau_{f}}\right)+R_{s}^{I}\exp\left(-\frac{t-t_{lm}}{\tau_{s}}\right)\right)$$ (11)

Where $t_{jk}$ is the timing of *k*-th spike on *j*-th neuron; $R^{E}$, $R_{f}^{I}$, $R_{s}^{I}$ denote relative amplitudes of excitatory, fast inhibitory and slow inhibitory currents, respectively (see **Table 2**). Time constants $\tau_{f}=0.5\;ms$ and $\tau_{f}=50\;ms$ define the time course of fast and slow post-synaptic currents, respectively. $E_{rev}^{E}=0\;mV$, $E_{rev}^{E}=-75mV$ are reversal potentials for excitatory and inhibitory currents, respectively. Synaptic multiplier $M_{ij}$ differentiates synaptic efficacy of inputs from specific neuron populations to others (see Table 3). The term $w_{ij}^{E}(t)$ indicates time-dependent synaptic weight changes driven by STDP between excitatory *i*-th and *j*-th neurons. $A_{ij}$ denotes terms in the adjacency matrix. $I_{syn}^{i}(t)$ is represented in$\;\frac{\mu A}{cm^{2}}$.

**Table 2:** Relative synaptic efficacies between the neuron types.

Synapse Type	Postsynaptic target neuron type	
	Excitatory	Inhibitory
$R^{e}$ (excitatory)	0.15	0.08
$R_{f}^{i}$ (fast inhibitory)	0	0.15
$R_{s}^{i}$ (slow inhibitory)	0.05	0

**Table 3:** Synaptic group multipliers. Intra-backbone connections are stronger than those between the SF neurons; the SF population receives stronger inhibitory input than backbone neurons.

$M_{ij}$		Postsynaptic		
		Backbone neurons	SF neurons	Inhibitory interneurons
**Presynaptic**	Backbone neurons	2.5	1	1
	SF neurons	1	1	1
	Inhibitory neurons	0.5	3.5	1

In a separate set of simulations (S2 Fig) we kept the strength of inhibitory connections to both SF and EB populations the same ($M_{ij}=2.5$ for both populations). Here, the $I_{drive}=2\frac{\mu A}{cm^{2}}$ for EB population and $I_{drive}=-0.5\frac{\mu A}{cm^{2}}$ for SF population.

For simulations that included STDP (Figs 3-5), the change in synaptic strength between postsynaptic neuron *i* and presynaptic neuron *j* was given by:


$$\Delta \omega_{ij}=\left\{ \begin{array}{cc} P_{x}A_{+}exp-\frac{\left|\Delta t_{ij}\right|}{\tau_{+}} & \Delta t_{ij}>0\\ {-P}_{x}A_{-}exp-\frac{\left|\Delta t_{ij}\right|}{\tau_{-}} & \Delta t_{ij}<0 \end{array}\right.\;$$ (12)

where *Δt*_*ij*_ is the difference between the most recent spike time of postsynaptic neuron *i* and presynaptic neuron *j*; $A_{+}$ and $A_{-}$ are the maximum rates of change (excluding $P_{x}$) with values $A_{+}=0.07$ and $A_{-}=0.025$. $\tau_{+}$ and $\tau_{-}$ are the time constants, $\tau_{+}=14$ ms and $\tau_{-}=34$ ms. The overall effect is plasticity that favors potentiation between neurons with more synchronous firing (Δt<~25 ms) and favors depression for longer inter-spike intervals. This leads to overall synaptic depression in a randomly and sparsely firing network, and to synaptic potentiation in a highly active network. $P_{x}$ is a network-wide controlled parameter for changing the rate of plasticity between specific neuronal groups (see Table 4). These parameters are set in a way that only excitatory-to-excitatory synapses undergo plastic changes, and the speed at which these changes occur is slower at inputs from the SF population than from backbone populations. Across intervals of STDP-associated plasticity, synaptic weight $w_{ij}$ for each connection is constrained to the interval [0, $w_{\max}$] where $w_{\max}=5.$ Initially synaptic weights were set to $\omega_{ij}(t=0)=1$ for most excitatory-to-excitatory connections, except for those of the EB 1 backbone targeting the SF population that receives strong connections from EB 1 only (i.e., blue group), where $\omega_{ij}(t=0)=1.2$. Similarly, $\omega_{ij}(t=0)=1.2$ for the EB 2 backbone synapses targeting the SF population that receives strong connections from EB 2 only (i.e., green group). We have tested the effects of this connection bias (S1 Fig) and observe that it has only minimal effect on both activation and segregation of the SF populations.

**Table 4:** Population-specific magnitude of synaptic plasticity. Plasticity of the synapses emanating from SF population is significantly slower than that of synapses originating from backbone population. Only excitatory-to-excitatory synapses undergo plastic changes.

$P_{x}$		Postsynaptic		
		Backbone neurons	SF neurons	Inhibitory interneurons
**Presynaptic**	Backbone neurons	0	1	0
	SF neurons	0.3	0.3	0
	Inhibitory interneurons	0	0	0

#### Memory testing: activity, synaptic strength, and functional connectivity analyses.

Average mean firing rates (**Figs 1**, **4 and 5**) were calculated for the SF population overall, or separately for each subgroup of the SF population described above. These measures were used to calculate mean “activation” and “segregation” of the SF population representation in EB 1 and EB 2. Activation of EB 1 vs. EB 2 (*A*_*1/2*_) is defined as $A_{1/2}=\frac{f_{1/2}-\tilde{f}}{f_{1/2}+\tilde{f}}$, where $f_{1/2}$ is the mean firing frequency of the green and blue SF groups during test bout activation of EB 1 and EB 2, and $\tilde{f}$ is the mean frequency of the non-activating SF neuron populations, reflective of the background noise (pink and purple SF). It measures the relative activation of the blue and green groups of SF neurons above background.

Activation of non-activating groups (pink and purple SF, Fig 5). Is calculated similarly as a difference in mean activation within the test bouts but compared to baseline test.

Segregation of EB 1 from EB 2 (S1/2) is defined as $S_{1/2}=\frac{\left|f_{1}-f_{2}\right|}{f_{1}+f_{2}}$, where $f_{1}$ ($f_{2}$) is the mean firing frequency of the blue and green SF cells during reactivation of EB 1 (EB 2), measuring the extent to which blue/green SF populations are differentially active in response to input the two backbones. A value near one indicates almost complete silence of one SF group while the other group is active, and vice versa. Segregation value is calculated separately for activation of both SF populations and then averaged.

Mean synaptic strengths of inputs from EB 1 and EB 2 backbone populations (x-axis and y-axis, respectively, demarked as ‘x’ on S4 Fig diagonal) were calculated for each SF subgroup defined during every test phase. Changes in mean synaptic strength between tests was calculated as a vector (Figs 4D, 4DD and S4 (off-diagonal)), by positioning the tail at the onset coordinates and head at the final coordinates. Synaptic strength change vectors were calculated between baseline and post-ACh^–^ sleep tests (or post-ACh^+^ sleep tests for reverse-order simulations), between post-ACh^–^ and post-ACh^+^ tests, and between baseline and post-ACh^–^→ACh^+^ tests (or post-ACh^+^→ACh^–^ tests for reverse-order simulations).

To quantify the pairwise functional connectivity between SF neurons (Figs 1E, 2B, 4E and 4EE) and from experimental recordings (Fig 2A), a metric based on the average minimal distance (AMD) was used [86]. The pairwise AMD between two neurons (*i, j*) was given by the mean difference of the time of each spike, *k* in spike train of the *i*-th neuron, *S*_*i*_, to the most recent preceding spike in the spike train of *j*-th neuron, *S*_*j*_. This was calculated as$,\;{AMD}_{ij}=\frac{1}{N_{i}}\sum_{k=1}^{N}{\Delta t}_{k}^{j}$, where *N*_*i*_ was the number of spikes in spike train *S*_*i*_ and ${\Delta t}_{k}^{j}$ was the temporal distance between *k*-th spike in *S*_*i*_ to the nearest spike in *S*_*j(151)*_. To quantify the magnitude of temporal locking between the pair of neurons independent of firing rate, a z-score was calculated, using *L* as the length of the inter-spike interval of spike train *S*_*i*_. The first and second moments ($\mu_{1}$ and $\mu_{2}$, respectively) for the spike train *S*_*i*_ are given by $\mu_{1}=\frac{1}{4T}\sum_{L}L^{2}\;and\;\mu_{2}=\frac{1}{12T}\sum_{L}L^{3}$, where *T* is the total time of the *S*_*i*_ -th spike train (in ms). These were used to derive mean and standard deviation of the minimal distance with respect to *S*_*i*_, where the mean $\mu =\mu_{1}$, the first moment, and the standard deviation $\sigma =\sqrt{\mu_{2}-\mu_{1}^{2}}$. The Z-score is given by $Z_{ij}=\frac{{AMD}_{ij}-\mu_{i}}{\sigma_{i}}$ (see [16,86]). Negative z-scores imply that two neurons fire in a less coincident fashion than random firing would instigate, indicating mutually inhibitory interactions, whereas positive z-scores imply coincident spiking above chance. Large magnitude z-scores indicate tight temporal coincidence.

To establish separation of two memories (Fig 4F and 4FF), the dot product of functional connectivity matrixes was calculated between testing periods when EB 1 vs. EB 2 backbone populations were reactivated. Data were averaged over 4 randomly-initialized simulations. Histograms of functional connectivity changes (S5 Fig) were calculated using the pairwise z-score distributions of every connection pair across compared test phases.

#### *In vivo* hippocampal recordings, and contextual fear conditioning (CFC).

Experimental methods are described in detail in [14,16]. Briefly, male C57BL/6J mice between 2 and 6 months (*n* = 4) were implanted with custom built driveable headstages with two bundles of stereotrodes implanted in bilateral CA1, and EMG electrodes to monitor nuchal muscle activity [14,147]. The signals from each of the stereotrode recording sites were split into local field potential (0.5-200 Hz) data, which were used to evaluate behavioral states, and spike data (200 Hz-8 kHz), which were used for functional connectivity analysis. Following post-operative recovery, mice underwent habituation to daily handling and tethered recording as stereotrodes were lowered into CA1 to obtain stable recordings. After establishing stable single-neuron recordings, each mouse underwent single-trial CFC (placement into a novel environmental context, followed 2.5 min later by a 2-s, 0.75 mA foot shock) starting at lights on (i.e., ZT 0), after which they were returned to their home cage for ad lib sleep with continued recording. Spike data from individual neurons was discriminated offline using standard methods (consistent waveform shape and amplitude on the two stereotrode wires, relative cluster position of spike waveforms in principle component space, ISI ≥ 1 ms) [14–16,18,19].

Following 24 h baseline recording, mice underwent either CFC [148] or Sham conditioning. Namely, mice were placed in a novel conditioning chamber and allowed to explore freely for either 150 s (CFC mice) or 180 s (Sham mice). CFC mice then received a 2s foot shock (0.75 mA), and were left in the conditioning chamber for an additional 28s. Following conditioning, mice were returned to their home cage in the sleep-recording chamber and underwent an additional 24 h period of undisturbed recording prior to contextual fear behavioral testing. Twenty-four hour following contextual fear or sham conditioning, mice were returned to the conditioning chamber for 5min, during which behavior was continuously video monitored. To quantify contextual fear conditioning, context-specific freezing was quantified as a change in the percentage of total recording time spent in stereotyped freezing behavior between the 5 min test period and the pre-shock interval in the initial training period.

Functional connectivity (Fig 2A) of neurons recorded from individual animals was calculated separately for each adjacent bout pair of NREM and REM sleep (REM onset needed to be < 10s following the NREM offset), using the AMD method described above. To offset length discrepancies between NREM and REM bouts affecting the statistical comparison, we analyzed only the final segment of NREM bout matching time duration of the paired (following) REM. The analysis was performed on both CFC and sham animals (i.e., no electrical shock during presentation of novel cage); 6h baseline was compared to 6h post experimental manipulation (i.e., CFC or sham).

## Supporting information

S1 Fig Tripling the population of all neurons reproduces the results from smaller networks. A) Differential activation of neural populations for ACh^-^ and ACh^+^ states (as in Fig1). B) recruitment and pruning of SF neurons during reactivation of two engram backbones (as in Fig 4).
(TIF)

S2 FigQualitatively same results in terms of differential activation of SF cells for ACh^+^ and ACh- states is obtained while keeping inhibition level to EB and SF populations the same.Synaptic multiplier, $M_{ij}=2.5$, for both populations; the $I_{drive}=2\frac{\mu A}{cm^{2}}$ for EB population and $I_{drive}=-0.5\frac{\mu A}{cm^{2}}$ for SF population.(TIF)

S3 FigActivation and segregation of memory 1 and memory 2 in SF layer as a function of connection multiplier, $\omega_{ij}\left(t=0\right)$ (see methods).When $\omega_{ij}(t=0)=1.0$ all SF groups have the same weight initially and the only difference between assigned SF subpopulations is a random fluctuation in the number of connections at baseline, from EB layer (as decribed in methods). Activity at baseline is measured before the sleep epochs occur. The multiplier $\omega_{ij}(t=0)$ does not affect significantly neighter activation or segregation of recruited SF neurons into memory 1 and memory 2.(TIF)

S4 FigConnection strength changes during simulated sleep memory consolidation.**DIAGONAL** representative connection maps from backbones of both memories to individual neurons in SF layer. Each dot represents a total connection strength (i.e., sum of synaptic efficacies of neurons belonging to one of the backbones and targeting given SF neuron) from backbone of EB 1 (X-axis) and EB 2 (Y-axis) to individual SF neurons. The connectivity is set at random. The whole population of SF layer at baseline is divided into 4 quartiles (and remains the same for the rest of the simulation): SF neurons receiving stronger input (i.e., more connections) from the memory 1 backbone population are shown in blue; those receiving stronger input (i.e., more connections) from the memory 2 backbone population only are shown in green. ***Pink*** and ***violet*** SF neurons indicate populations receiving weak (i.e., least connections from both backbones) and strong input from both backbone populations (i.e., most connections from both backbones), respectively. During most of the simulations (except **S5 Fig**) these groups receive lower constant current ($I_{drive}$) than ***green*** and ***blue*** SF groups, which leads to reduced, more random, firing patterns. **Top left**: representative map obtained at baseline. **Center**: representative map obtained post-ACh-. **Bottom right:** representative connection map obtained post-ACh^–^→ACh^+^. X - denotes mean connection strength for the given population. OFF-DIGONAL Change of mean connection strength between following timepoint tests: baseline to post-ACh^-^, post-ACh^-^ to post-ACh^+^, baseline to post-ACh^–^→ACh^+^. Values indicate mean values of 4 simulation runs.(TIF)

S5 Fig ***A)* Histograms of pairwise functional connectivity changes between the SF neurons after ACh**^**-**^**, after ACh**^**+**^**, and after a ACh**^**–**^**→ACh**^**+**^ **cycle.** ACh^-^ state indiscriminately recruits SF neurons into the engram by strengthening connections from backbone neurons to SF neuron populations. This results in a shift of the functional connectivity distributions towards stronger connections (higher z-scores) within the SF neuron population (left). ACh^+^ prunes these connections, leaving/strengthening only the strongest ones, shifting the distribution towards lower functional connectivity values (lower z-scores) and compared to ACh^-^ (center). When an ACh^-^ state is followed by an ACh^+^ state (right), a small group of functional connections are especially strengthened, illustrative of recruitment of some SF cells into the engram. ***B)* Histograms of pairwise functional connectivity changes between the SF neurons after ACh**^**+**^**, after ACh**^**-**^**, and after a (reversed) ACh**^**+**^**→ACh**^**–**^ **state cycle (i.e., REM precedes NREM)** Because ACh^+^ prunes backbone of SF connections before SF cells could be recruited into the engram, there are no significant shifts in the distributions of the connections.
(TIF)

S6 Fig Activation and segregation of the recruited SF representations as a function of length of reactivation bouts of memory 1 and memory 2 backbones. Reactivation bouts were varied between 200–900 ms. ***A)*** Sample raster for 200 ms reactivation bouts. ***B)*** Sample raster for 800 ms reactivation bout. ***C)*** Calculation of activation and segregation (see **Methods**) as a function of length of reactivation bouts. Both activation and segregation are largely independent of reactivation bout length. Results in ***C*** averaged over 5 simulation runs.
(TIF)

S7 Fig Activation and separation of the recruited SF representations as a function of activation delay between reactivation bouts of memory 1 and memory 2 backbones. The reactivation bout duration is set to 200 ms. The reactivation delay (i.e., dead space between two consecutive reactivations) is varied between 0–800 ms. ***A)*** Sample raster of simulation with an activation delay of 200 ms. ***B)*** Activation and segregation of SF representations (see **Methods**) as a function of length of reactivation delay. Both activation and segregation decrease as a function of activation delayed. However, when the simulation length is controlled for total reactivation time (which decreases as a function of reactivation delay), both functions recover. This indicates that total reactivation time controls activation and segregation magnitude rather than reactivation delay. Values in ***B*** indicate mean of 5 simulation runs.
(TIF)

S8 FigActivation and segregation of SF neurons belonging to EB 1/EB 2 as a function of activation of neurons shared by both memories (i.e., SF sub-population that has largest number of connections with backbones of both memories; group denoted as violet on Figs 4, 5, S2, S4, and S5).To activate this sub-population of SF cells, we changed its $I_{Drive}$value from that of the other two (i.e., ***green*** and ***blue***) SF populations; invariably, $I_{Drive}$=-6$\frac{\mu A}{cm^{2}}$ for the population having the least connections with the backbones of the two memories (i.e., group dentoted as pink **on Figs 4, 5, S2, S4, and S5**). The x-axis denotes difference in $I_{Drive}$ between the violet and green/blue SF population. When the $I_{Drive}$ is the same for all the groups the ***common subpopulation*** activates strongly (**violet line**), as do both memories, EB 1 and EB 2 (***blue*** line). The segregation (**orange line**) is however impeded. As the SF common block population is progressively inactivated (larger values of $I_{Drive}$difference) the segregation returns to normal levels. This intuitively indicates that if the memories share large common engram population the memories cannot be segregated and a single consolidated engram forms.(TIF)

S9 Fig Bias in one of the engrams leads to its preferential consolidation. EB 2 (denoted as green) receives longer reactivation (A) or stronger reactivation in terms of cell activation (B). In both cases EB 2 consolidates preferentially to show increased activation than EB 1 during post-test. A) EB1 (blue) is reactivated for 200ms per cycle whereas EB 2 is reactivated for 400ms per cycle. B) Weaker reactivation of one of the memories (lower constant current drive (I_Drive_)).
(TIF)
