# Supplementary figures and images for: Cholinergic modulation of neural networks supports sequential and complementary roles for NREM and REM states in memory consolidation
Source: PLoS Comput Biol. 2025 Jun 17;21(6):e1013097. doi: 10.1371/journal.pcbi.1013097 (PMC12201672; doi:10.1371/journal.pcbi.1013097)

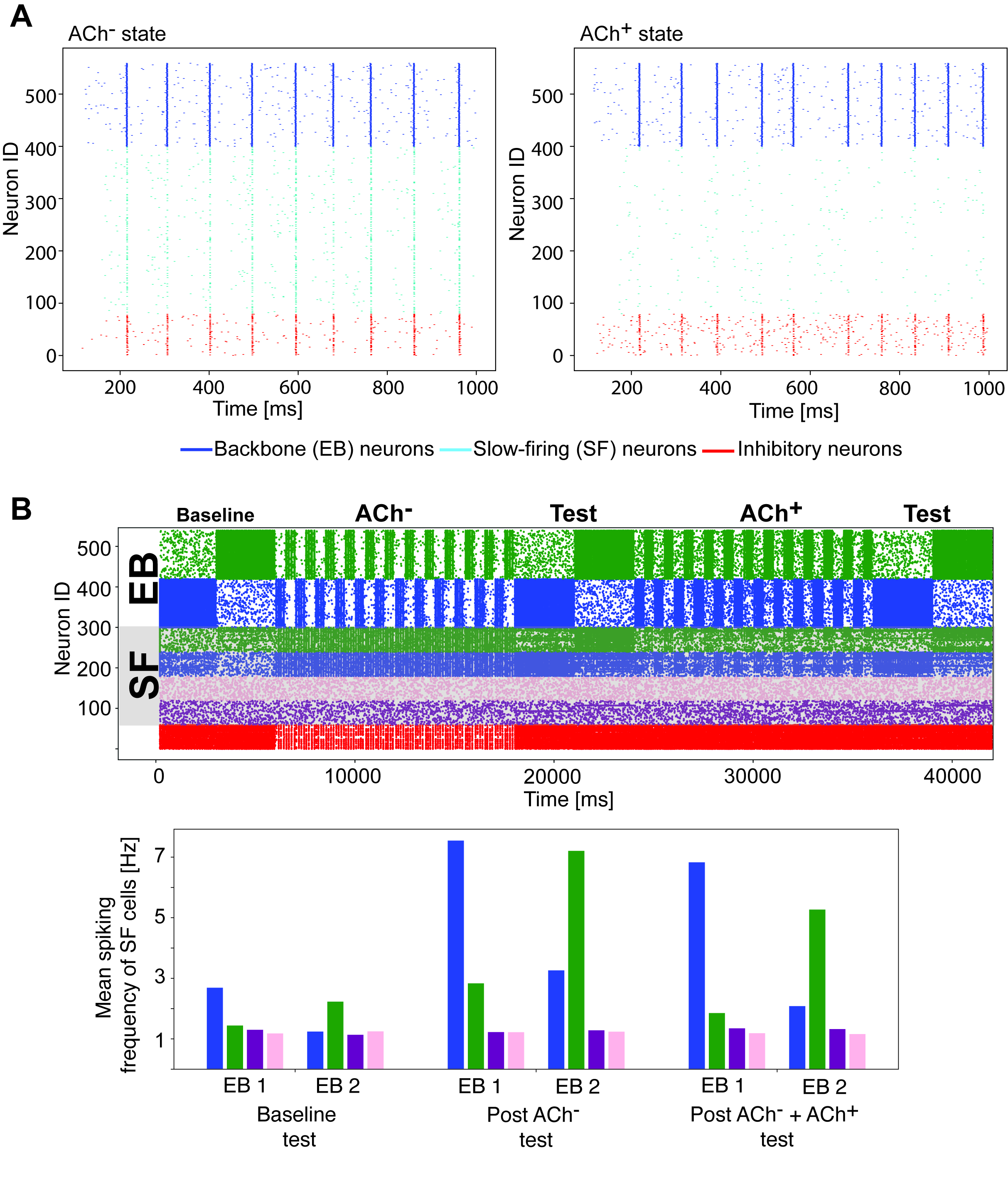

Supplement: S1 Fig — A) Differential activation of neural populations for ACh- and ACh+ states (as in Fig1). B) recruitment and pruning of SF neurons during reactivation of two engram backbones (as in Fig 4). (TIF) [file pcbi.1013097.s001.tif]

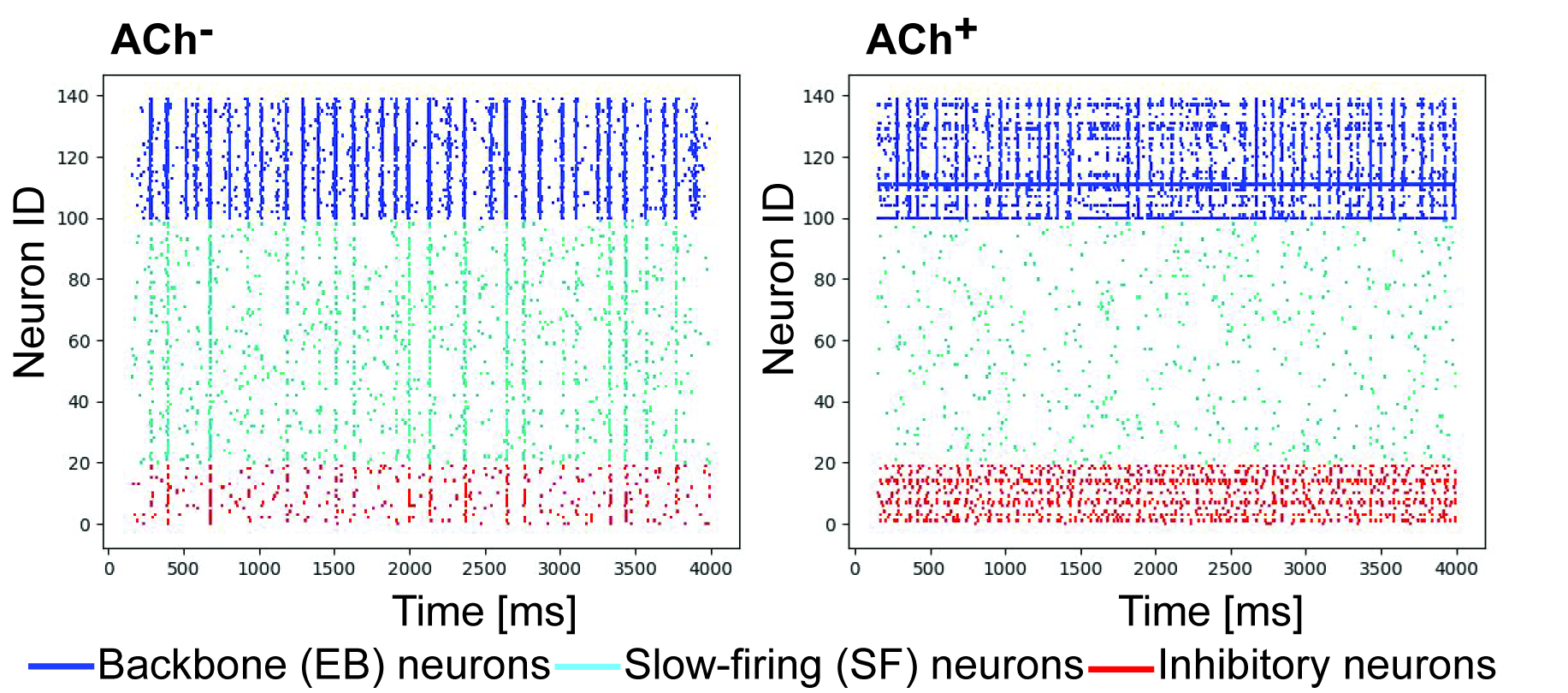

Supplement: S2 Fig — Synaptic multiplier, Mij=2.5, for both populations; the Idrive=2μAcm2 for EB population and Idrive=−0.5μAcm2 for SF population. (TIF) [file pcbi.1013097.s002.tif]

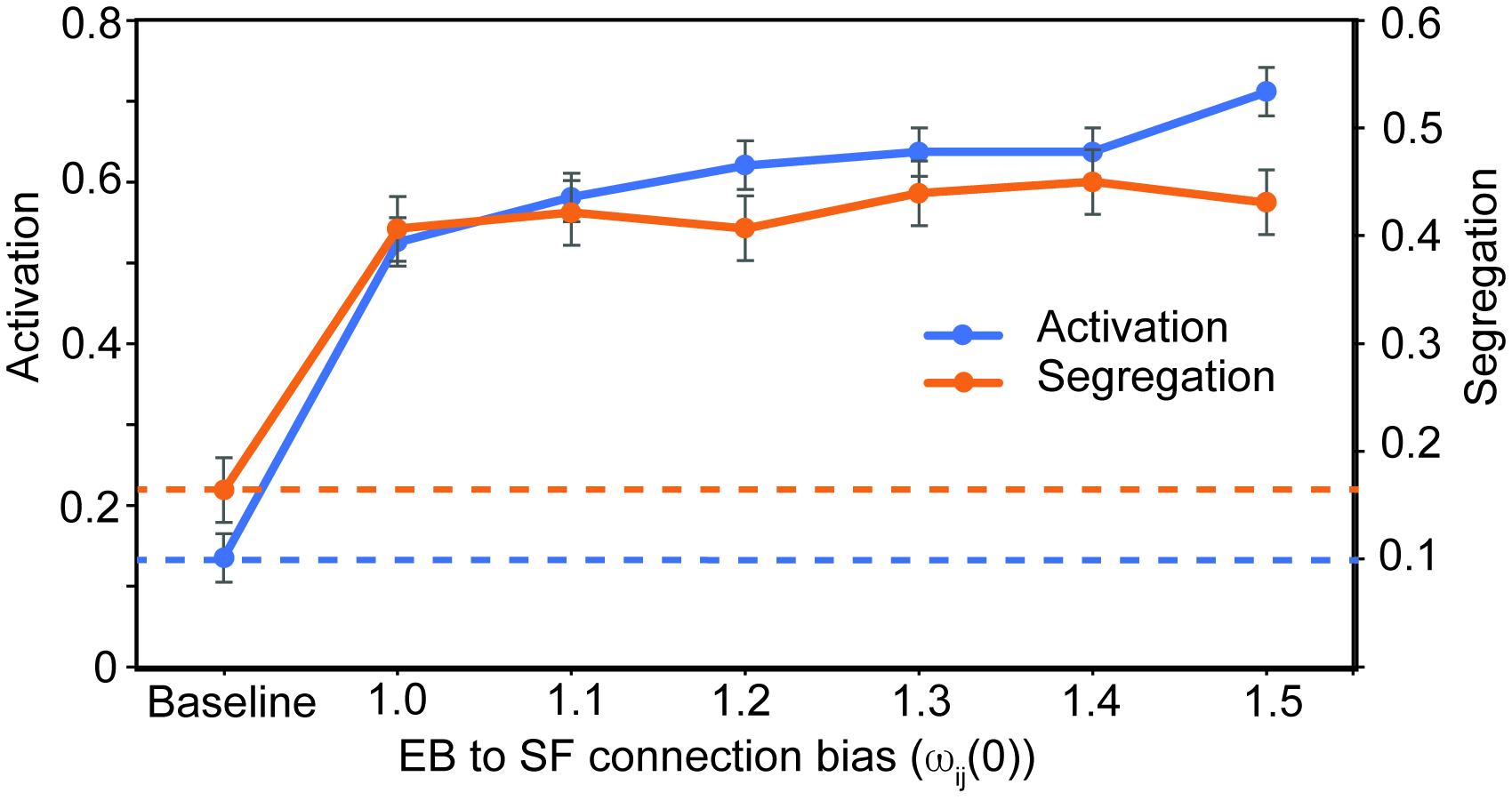

Supplement: S3 Fig — When ωij(t=0)=1.0 all SF groups have the same weight initially and the only difference between assigned SF subpopulations is a random fluctuation in the number of connections at baseline, from EB layer (as decribed in methods). Activity at baseline is measured before the sleep epochs occur. The multiplier ωij(t=0) does not affect significantly neighter activation or segregation of recruited SF neurons into memory 1 and memory 2. (TIF) [file pcbi.1013097.s003.tif]

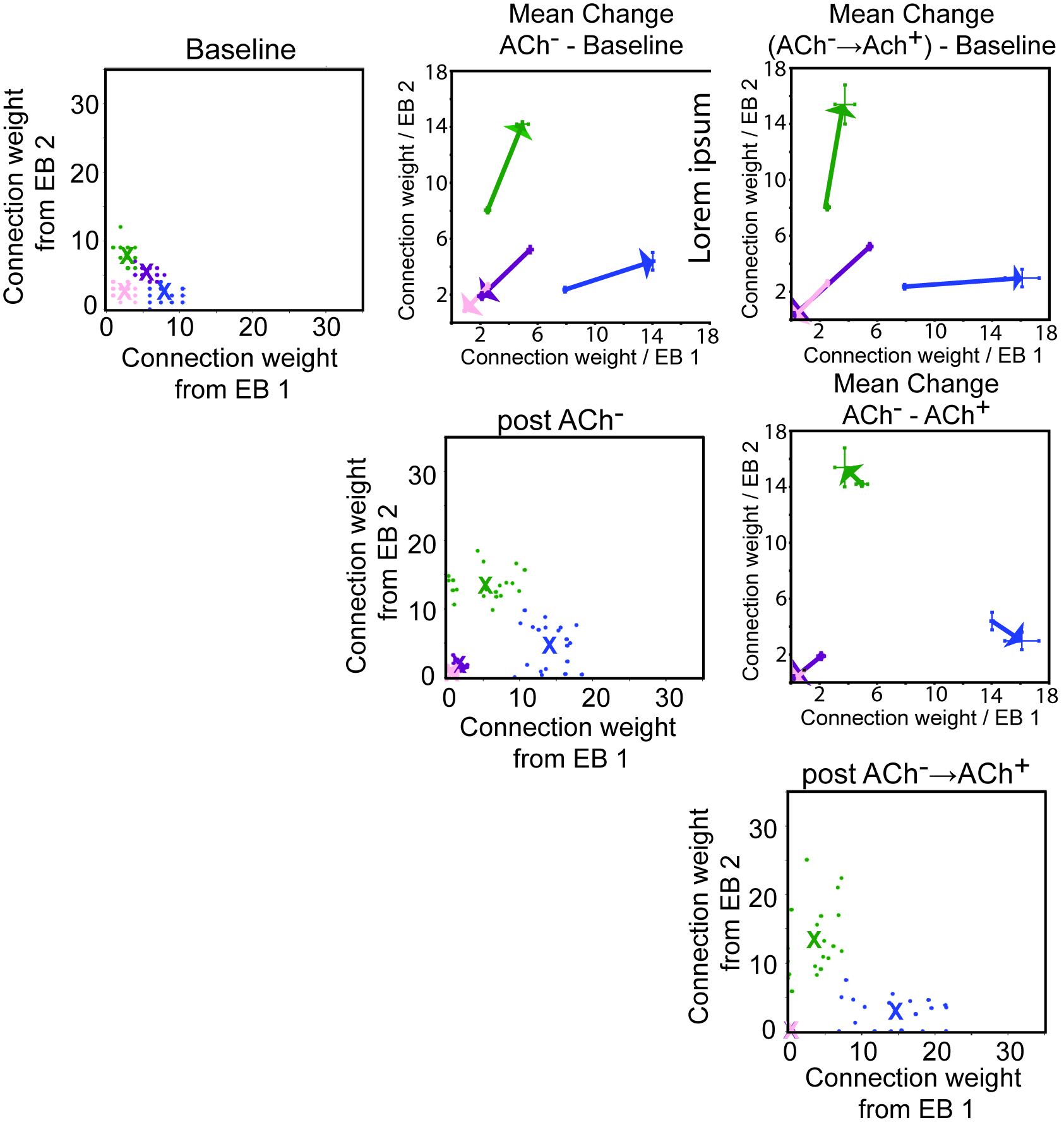

Supplement: S4 Fig — DIAGONAL representative connection maps from backbones of both memories to individual neurons in SF layer. Each dot represents a total connection strength (i.e., sum of synaptic efficacies of neurons belonging to one of the backbones and targeting given SF neuron) from backbone of EB 1 (X-axis) and EB 2 (Y-axis) to individual SF neurons. The connectivity is set at random. The whole population of SF layer at baseline is divided into 4 quartiles (and remains the same for the rest of the simulation): SF neurons receiving stronger input (i.e., more connections) from the memory 1 backbone population are shown in blue; those receiving stronger input (i.e., more connections) from the memory 2 backbone population only are shown in green. Pink and violet SF neurons indicate populations receiving weak (i.e., least connections from both backbones) and strong input from both backbone populations (i.e., most connections from both backbones), respectively. During most of the simulations (except S5 Fig) these groups receive lower constant current (Idrive) than green and blue SF groups, which leads to reduced, more random, firing patterns. Top left: representative map obtained at baseline. Center: representative map obtained post-ACh-. Bottom right: representative connection map obtained post-ACh–→ACh+. X - denotes mean connection strength for the given population. OFF-DIGONAL Change of mean connection strength between following timepoint tests: baseline to post-ACh-, post-ACh- to post-ACh+, baseline to post-ACh–→ACh+. Values indicate mean values of 4 simulation runs. (TIF) [file pcbi.1013097.s004.tif]

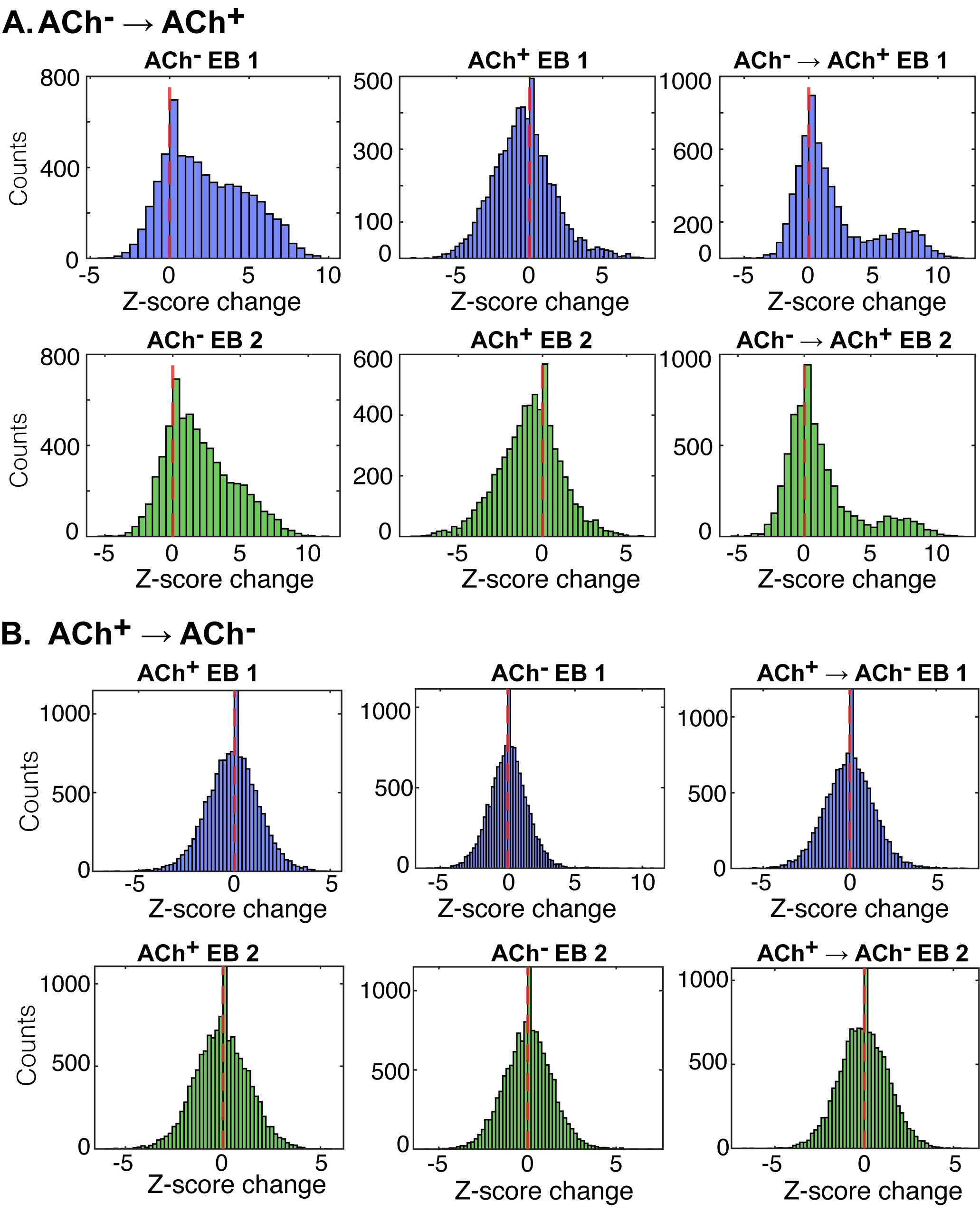

Supplement: S5 Fig — A) Histograms of pairwise functional connectivity changes between the SF neurons after ACh-, after ACh+, and after a ACh–→ACh+ cycle. ACh- state indiscriminately recruits SF neurons into the engram by strengthening connections from backbone neurons to SF neuron populations. This results in a shift of the functional connectivity distributions towards stronger connections (higher z-scores) within the SF neuron population (left). ACh+ prunes these connections, leaving/strengthening only the strongest ones, shifting the distribution towards lower functional connectivity values (lower z-scores) and compared to ACh- (center). When an ACh- state is followed by an ACh+ state (right), a small group of functional connections are especially strengthened, illustrative of recruitment of some SF cells into the engram. B) Histograms of pairwise functional connectivity changes between the SF neurons after ACh+, after ACh-, and after a (reversed) ACh+→ACh– state cycle (i.e., REM precedes NREM) Because ACh+ prunes backbone of SF connections before SF cells could be recruited into the engram, there are no significant shifts in the distributions of the connections. (TIF) [file pcbi.1013097.s005.tif]

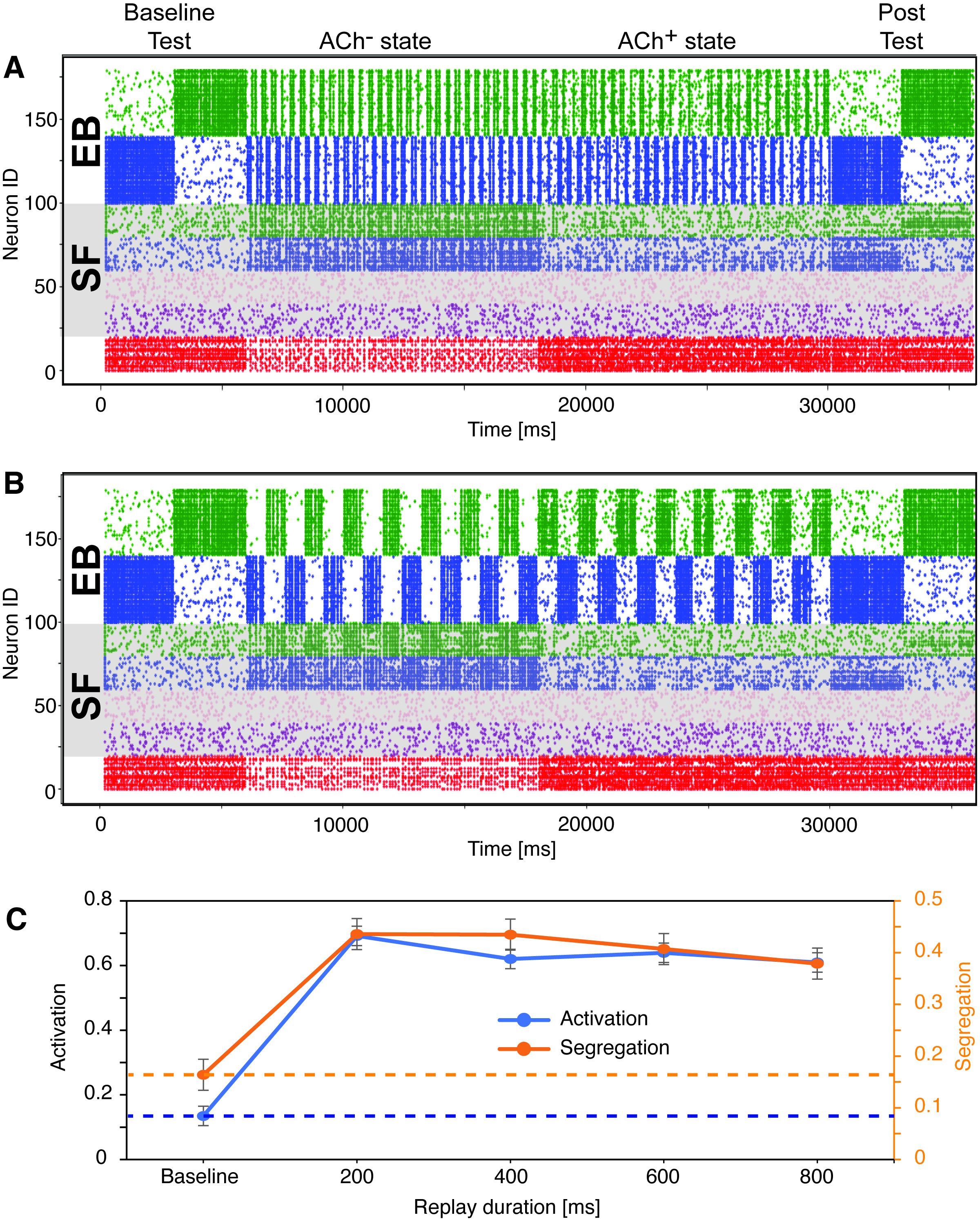

Supplement: S6 Fig — Reactivation bouts were varied between 200–900 ms. A) Sample raster for 200 ms reactivation bouts. B) Sample raster for 800 ms reactivation bout. C) Calculation of activation and segregation (see Methods) as a function of length of reactivation bouts. Both activation and segregation are largely independent of reactivation bout length. Results in C averaged over 5 simulation runs. (TIF) [file pcbi.1013097.s006.tif]

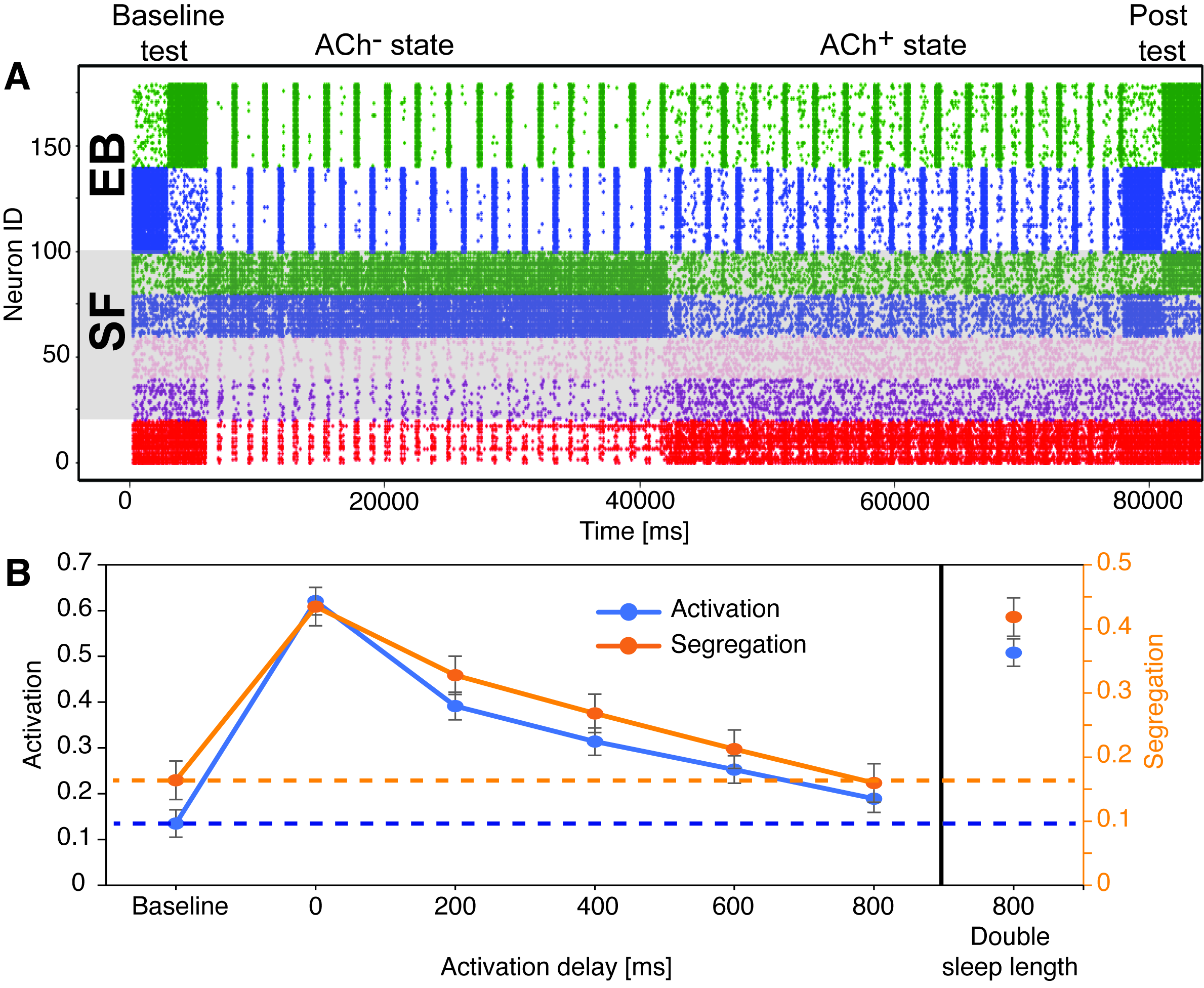

Supplement: S7 Fig — The reactivation bout duration is set to 200 ms. The reactivation delay (i.e., dead space between two consecutive reactivations) is varied between 0–800 ms. A) Sample raster of simulation with an activation delay of 200 ms. B) Activation and segregation of SF representations (see Methods) as a function of length of reactivation delay. Both activation and segregation decrease as a function of activation delayed. However, when the simulation length is controlled for total reactivation time (which decreases as a function of reactivation delay), both functions recover. This indicates that total reactivation time controls activation and segregation magnitude rather than reactivation delay. Values in B indicate mean of 5 simulation runs. (TIF) [file pcbi.1013097.s007.tif]

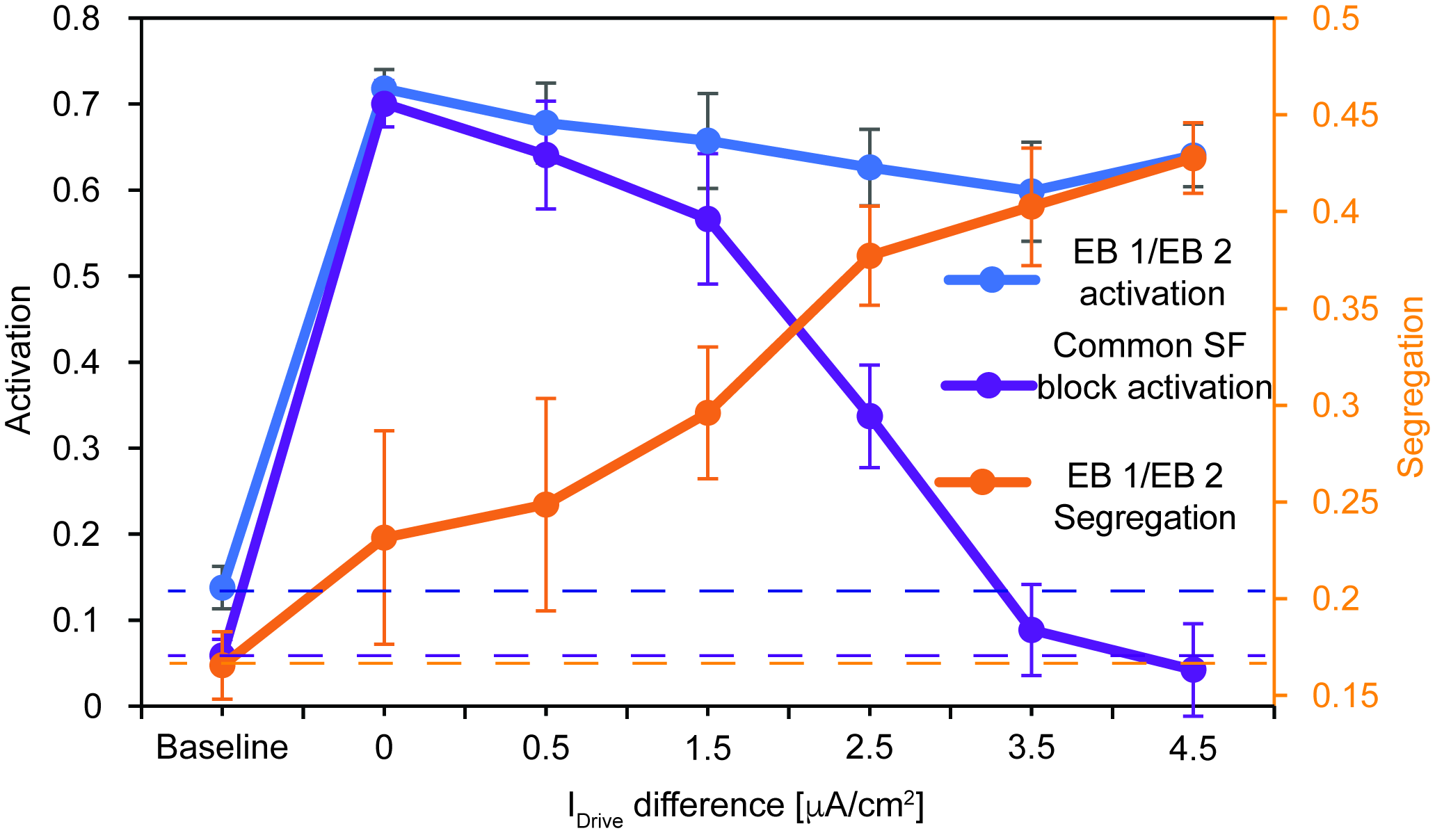

Supplement: S8 Fig — To activate this sub-population of SF cells, we changed its IDrivevalue from that of the other two (i.e., green and blue) SF populations; invariably, IDrive=-6μAcm2 for the population having the least connections with the backbones of the two memories (i.e., group dentoted as pink on Figs 4, 5, S2, S4, and S5). The x-axis denotes difference in IDrive between the violet and green/blue SF population. When the IDrive is the same for all the groups the common subpopulation activates strongly (violet line), as do both memories, EB 1 and EB 2 (blue line). The segregation (orange line) is however impeded. As the SF common block population is progressively inactivated (larger values of IDrivedifference) the segregation returns to normal levels. This intuitively indicates that if the memories share large common engram population the memories cannot be segregated and a single consolidated engram forms. (TIF) [file pcbi.1013097.s008.tif]

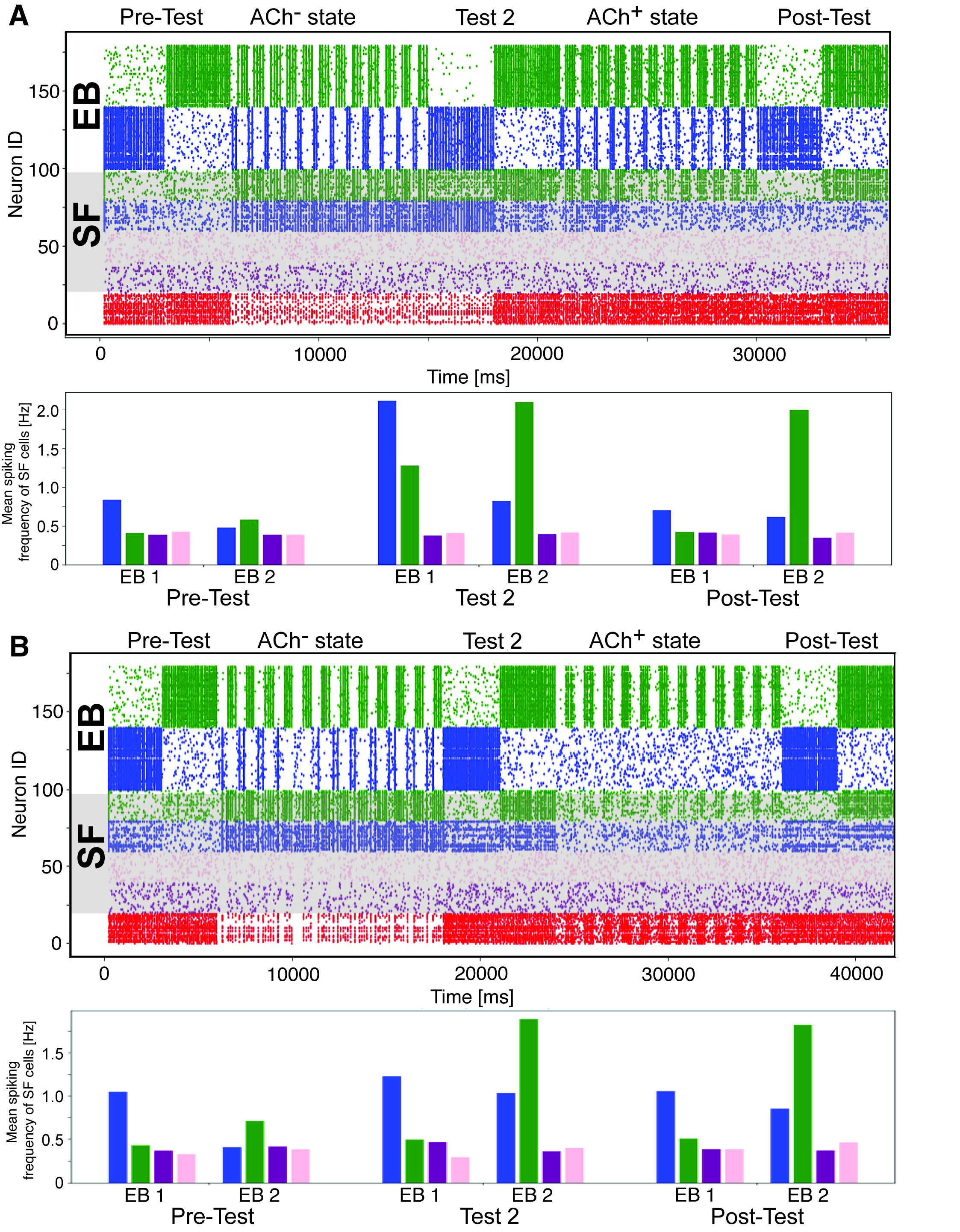

Supplement: S9 Fig — EB 2 (denoted as green) receives longer reactivation (A) or stronger reactivation in terms of cell activation (B). In both cases EB 2 consolidates preferentially to show increased activation than EB 1 during post-test. A) EB1 (blue) is reactivated for 200ms per cycle whereas EB 2 is reactivated for 400ms per cycle. B) Weaker reactivation of one of the memories (lower constant current drive (IDrive)). (TIF) [file pcbi.1013097.s009.tif]
